# Supplementary material for: c-MYC-Induced Sebaceous Gland Differentiation Is Controlled by an Androgen Receptor/p53 Axis
Source: Cell Rep. 2013 Feb 21;3(2):427–41. doi: 10.1016/j.celrep.2013.01.013 (PMC3778892; doi:10.1016/j.celrep.2013.01.013)
Supplement: Table S1. Primary Antibodies Used [file mmc1.pdf]

Table S1. Primary Antibodies Used

Species Reactivity in this paper	Antibody	Version/Clone	Supplier	Catalogue Number	Host Species	IF Dilution	IHC Dilution
Mouse	anti-AR	(N-20)	SCBT	sc-816	Rabbit	1:50	1:750
Mouse	anti-AR	(ΔN1-15)	Thermo Scientific Pierce	MA1150	Rat	1:50 when diluted to 1mg/ml stock	
Human (Not Mouse)	anti-AR	(AR27)	Novocastra	NCL-AR-318	Mouse		1:50
Mouse/Human	anti-c-MYC		Millipore	06-340	Rabbit	1:50	1:50-1:200
Mouse	anti-FASN	(G-11)	SCBT	sc-48357	Mouse	1:100	1:50 m.o.m
Mouse	anti-p53	(CM5)	Novocastra	P53-CM5P	Rabbit		1:200
Human (Not Mouse)	anti-p53	(DO-7)	Dako	M7001	Mouse		1:1000
Mouse	anti-PPARγ	(E-8)	SCBT	sc-7273	Mouse	1:100	
Mouse	anti-PCNA	(C-20)	SCBT	sc-9857	Goat	1:100	
Mouse	anti-γH2AX	(JBW301)	Millipore	05-636	Mouse	1:500	
Mouse	anti-Involucrin (IVL)	(ERLI-3)	CRUK	in house	Rabbit	1:200	
Mouse	anti-BLIMP1	(6D3)	eBiosciences	14-5963	Rat	1:50	
Mouse	anti-Ki67	(Tec-3)	Dako	M7249	Rat	1:100	1:500
Mouse	anti-Ki67	(SP6)	Vector Labs	VP-RM04	Rabbit		1:100
Mouse	Anti-ERα	(MC-20)	SCBT	sc-542	Rabbit		1:50

IF: immunofluorescence staining; IHC: immunohistochemical staining; m.o.m: mouse on mouse.
