# Supplementary material for: c-MYC-Induced Sebaceous Gland Differentiation Is Controlled by an Androgen Receptor/p53 Axis
Source: Cell Rep. 2013 Feb 21;3(2):427–41. doi: 10.1016/j.celrep.2013.01.013 (PMC3778892; doi:10.1016/j.celrep.2013.01.013)
Supplement: Document S1. Article plus Supplemental Information [file mmc2.pdf]

Cell Reports
Article

Cell PRESS
Open ACCESS

c-MYC-Induced Sebaceous Gland Differentiation Is Controlled by an Androgen Receptor/p53 Axis

Denny L. Cottle,[1] Kai Kretzschmar,[1,5] Pawel J. Schweiger,[4] Sven R. Quist,[2,4] Harald P. Gollnick,[2] Ken Natsuga,[3,4] Satoru Aoyagi,[3] and Fiona M. Watt[1,4,5,*]
[1]Wellcome Trust-Medical Research Council Stem Cell Institute (SCI), University of Cambridge, Tennis Court Road, Cambridge CB2 1QR, UK
[2]Clinic of Dermatology and Venereology, Otto-von-Guericke University, Magdeburg, Leipziger Str. 44, DE-39120 Magdeburg, Germany
[3]Department of Dermatology, Hokkaido University School of Medicine, North 15 West 7, Sapporo 060-8638, Japan
[4]Cancer Research UK Cambridge Research Institute (CRI), Li Ka Shing Centre, Cambridge CB2 0RE, UK
[5]Centre for Stem Cells and Regenerative Medicine, King's College London, 28th Floor, Tower Wing, Guy's Hospital, Great Maze Pond, London SE1 9RT, UK
*Correspondence: fiona.watt@kcl.ac.uk
http://dx.doi.org/10.1016/j.celrep.2013.01.013

SUMMARY

Although the sebaceous gland (SG) plays an important role in skin function, the mechanisms regulating SG differentiation and carcinoma formation are poorly understood. We previously reported that c-MYC overexpression stimulates SG differentiation. We now demonstrate roles for the androgen receptor (AR) and p53. MYC-induced SG differentiation was reduced in mice lacking a functional AR. High levels of MYC triggered a p53-dependent DNA damage response, leading to accumulation of proliferative SG progenitors and inhibition of AR signaling. Conversely, testosterone treatment or p53 deletion activated AR signaling and restored MYC-induced differentiation. Poorly differentiated human sebaceous carcinomas exhibited high p53 and low AR expression. Thus, the consequences of overactivating MYC in the SG depend on whether AR or p53 is activated, as they form a regulatory axis controlling proliferation and differentiation.

INTRODUCTION

The sebaceous gland (SG) is part of the epidermis and produces the sebum that lubricates the skin surface. SGs are often associated with hair follicles (HFs), and loss of sebocyte function can lead to scarring alopecia, indicating a dependence of HFs on SGs (Sundberg et al., 2000). SG products also form the protective lipid barrier of the skin and thus function in skin immunity (Schneider and Paus, 2010), whereas specialized eyelid SGs (meibomian glands) provide a lipid film to prevent drying of the eye surface (McCulley and Shine, 2004). Sebaceous carcinomas, although rare, often recur locally, frequently metastasize, and have high mortality (Buitrago and Joseph, 2008). There is therefore considerable interest in elucidating SG biology.

Myc was first identified as the cellular homolog of the avian myelocytomatosis viral oncogene (*v-Myc*) (Sheiness et al., 1980) and originally designated *c-Myc* for cellular-Myc (Hayward et al., 1981). MYC is a transcription factor that regulates sebocyte differentiation (Arnold and Watt, 2001; Honeycutt and Roop, 2004; Watt et al., 2008). *K14MycER* transgenic mice provide an experimental model with which to study MYC-regulated sebocyte differentiation. In these mice, human MYC is expressed under the control of the human keratin 14 (*K14*) promoter as a fusion protein with a mutant mouse estrogen receptor (ER) α ligand-binding domain. The *MycER* transgene is constitutively expressed in the basal layer of the epidermis, but MYCER protein (henceforth MYC) is only activated upon topical activation of 4-hydroxytamoxifen (4OHT). Low levels of MYC activation promote SG expansion and differentiation, whereas high levels stimulate SG proliferation and inhibit differentiation (Berta et al., 2010). MYC is often associated with histone modifications marking active genes (Nascimento et al., 2011; Rahl et al., 2010) and may therefore serve to amplify the program of gene expression dictated by other transcription factors. These observations led us to explore potential factors that influence the outcome of MYC activation in the SG.

The *Ar* has been identified by chromatin immunoprecipitation (ChIP) as a MYC target gene in mouse epidermis (Nascimento et al., 2011), and MYC can promote androgen receptor (AR) activity and *AR* mRNA expression in human prostate (Nadiminty et al., 2012). In rats and humans, the AR is an early marker of sebocyte differentiation (Bayer-Garner et al., 1999; Rosenfield et al., 1998). In primary rat preputial sebocyte cultures, androgen inhibits proliferation (Deplewski and Rosenfield, 1999). In cultured human sebocytes, androgen can either promote or inhibit proliferation, depending on the type of androgen and the origin of the cells (Akamatsu et al., 1992). In vivo androgens can promote growth and development of the human sebaceous gland (Zouboulis, 2010), while poorly differentiated sebaceous carcinomas have reduced AR expression (Bayer-Garner et al., 1999). Nevertheless, testicular feminization (TFM) mice, which have a spontaneous loss of function *Ar* mutation (Gaspar et al., 1991), still form SGs (Markova et al., 2004), suggesting the AR is dispensable for morphogenesis.

Another candidate modulator of MYC activity is p53. p53 is mutated in two-thirds of sebaceous carcinomas (Kiyosaki et al., 2010). Oncogenic levels of MYC activity promote p53 activation indirectly because of DNA damage (Hong et al.,

CrossMark

2006; Murphy et al., 2008; Pusapati et al., 2006). In human sebaceous carcinomas that retain p53 expression, elevated p53 protein levels correlate with worse prognostic outcome (Hasebe et al., 1994; Hayashi et al., 1994; Izumi et al., 2008).

In the prostate, a mutually antagonistic relationship exists between the AR and p53. p53 can inhibit *AR* gene expression by direct association with the AR promoter (Alimirah et al., 2007) and by inhibiting AR protein activity (Shenk et al., 2001). Conversely, strong AR activity can inhibit *p53* expression (Rokhlin et al., 2005) and p53 activity (Nantermet et al., 2004). Several of the p53 mutants identified in sebaceous carcinoma (Kiyosaki et al., 2010) are also found in prostate cancer and retain the ability to impair AR signaling, despite being mutations in the DNA-binding domain (Nesslinger et al., 2003).

Collectively, these observations prompted us to explore the role of AR and p53 in modulating the consequences of MYC activation in the sebaceous gland.

RESULTS

Markers of SG Differentiation

The SG is a sac-like structure, comprising an undifferentiated, proliferating, peripheral (basal) layer that gives rise to centrally located differentiating sebocytes. As they accumulate lipids, differentiating sebocytes increase in size (Montagna et al., 1963; Rosenfield, 1989), eventually bursting to release their contents into the sebaceous duct (SD), a thin, cornifying squamous epithelium that connects the SG to the infundibulum of the HF (Laurent et al., 1992; Schneider and Paus, 2010). Experimental evidence from DNA label retaining cells (Reichelt et al., 2004) and lineage tracing has established the existence of SG stem cells (SCs) in the upper HF (Jensen et al., 2009; Snippert et al., 2010). Their progeny travel around the basal layer to the lower sebaceous tip before transiting internally and upward toward the SD as they undergo terminal differentiation (Cui et al., 2003; Petersson et al., 2011) (Figure 1N).

In telogen back skin of wild-type (WT) mice (Figures 1A–1M), strong nuclear AR was observed in the lower SG, with weak cytoplasmic and nuclear AR in the upper SG and elsewhere in the epidermis (Figures 1A and 1B). Proliferative SG cells, marked by Ki67 and proliferating cell nuclear antigen (PCNA) expression, were enriched near the basal SG tip and had weak nuclear AR (Figures 1C and 1D). Nuclear AR is indicative of active AR signaling, and although the distribution of AR-positive cells in the SG was the same in the males and females, males tended to exhibit stronger nuclear staining than females (Figure 1B) (Azzi et al., 2006). Skin is also a local source of androgens (Chen et al., 2006, 2010). Antibody specificity was confirmed by staining skin of *AR-TFM* mice, which lack functional ARs (Figures S1N, S1O, and S1P).

Differentiating sebocytes expressed lipogenic enzymes, such as fatty acid synthase (FASN) and peroxisome proliferator activated receptor γ (PPARγ) (Figures 1E and 1F). The largest sebocytes, located next to the SD, had low AR activity, expressed the *Myc* corepressor, B-lymphocyte induced maturation protein (BLIMP1) (Figure 1G), and were weakly positive for involucrin (IVL) (Lo Celso et al., 2008; Zouboulis et al., 1998) (Figure 1H). Although BLIMP1 is reported to be a sebocyte progenitor marker (Horsley et al., 2006), we only observed BLIMP1 expression in differentiating sebocytes (Magnúsdóttir et al., 2007) and in keratinocytes of the SD, marked by IVL (Latham et al., 1989) (Figure 1H, see arrows). This is consistent with previous reports that BLIMP1 is a marker of terminal differentiation in all epidermal lineages (Chang et al., 2002; Lo Celso et al., 2008; Magnúsdóttir et al., 2007; Sellheyer and Krahl, 2010).

In wild-type mouse skin, MYC expression was higher in the interfollicular epidermis (IFE) and SG than HFs (Reichelt et al., 2004), consistent with the finding that MYC activation favors differentiation along these lineages (Arnold and Watt, 2001; Honeycutt and Roop, 2004) (Figure 1J). MYC was expressed by differentiating, AR-positive sebocytes (detected with a 1:50 antibody dilution) (Figure 1K); however, the SG cells with highest levels of MYC (detected with a 1:200 antibody dilution) were basal, proliferating cells with low levels of AR (Figures 1L and 1M; Figure S1). Antibody specificity was confirmed on *K14MycER* and epidermal *Myc* knockout mice (Figures S1B–S1G, S1L, and S1M).

Figure 1N summarizes marker expression within the SG. Proliferative basal cells express the highest levels of MYC, whereas early differentiating sebocytes at the base of the SG have high levels of nuclear AR. Mid-differentiated cells exhibit high nuclear AR and express high levels of FASN and PPARγ. Late-stage differentiating sebocytes, in the upper part of the gland, exhibit low levels of AR activity, low expression of IVL, FASN, and PPARγ, and are BLIMP1+ve.

Effect of MYC on AR Activity

We next examined the effect of MYC activation on proliferation, differentiation, and AR activity in the epidermis. *K14MycER* mice were treated once with a low (0.1 mg) or high (1.5 mg) dose of 4OHT or vehicle (acetone) and examined for up to 8 days (Figure S2A). Control *K14MycER* mice treated with acetone and WT mice treated with 1.5 mg 4OHT were indistinguishable from untreated WT mice (Figures 2A–2G) (Arnold and Watt, 2001; Berta et al., 2010). Although MYC activation is known to promote IFE thickening and to cause hair follicle abnormalities in *K14MycER* mice (Arnold and Watt, 2001), lineage tracing established that there was no relocation of cells from the IFE to the SG (data not shown).

Four days following low-dose 4OHT treatment, the SG of *K14MycER* mice was enlarged and the differentiation compartment expanded, as assessed by hematoxylin and eosin staining (H&E) staining (Figure S2B) and AR and FASN expression (Figures 2D–2I). On activation, the AR translocates to the nucleus, and this provides a readout of AR activity in vivo. A secondary readout is expression of FASN, which is an AR-responsive gene (Schirra et al., 2005). Differentiation was quantified (Figure 2C) by measuring the average cross-sectional area of the SG differentiation compartment (Figure S2B).

The SGs of *K14MycER* mice treated with 0.1 mg 4OHT enlarged by 4 days. This was not due to increased cell size (Figure 2J) but to an increased number of differentiating sebocytes (Figure 2K), including AR-positive cells (bracket, Figure 2I), which correlated with increased proliferation predominantly in basal layer cells, but also occasionally in differentiated cells. By 8 days, low-dose 4OHT-treated SGs were hyperplastic

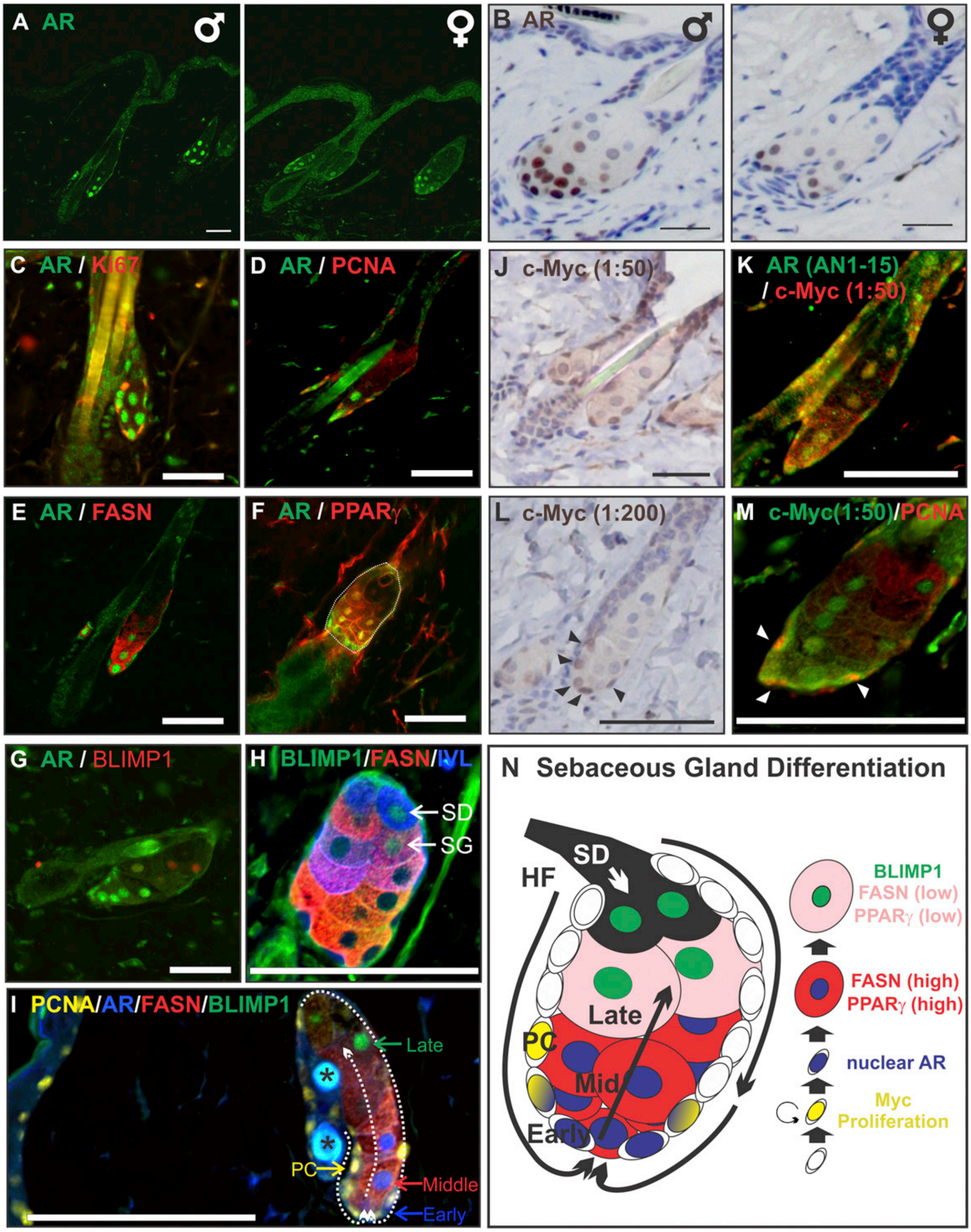

Figure 1. Expression of the AR and Other Markers in the SG

(A–M) Adult WT mouse telogen back skin labeled with antibodies to the markers indicated. (H) Arrows indicate a cell of either sebaceous duct (SD) or sebaceous gland lineage (SG). (J–M) c-MYC antibody dilution is indicated. SG is outlined by dashed line in (F). Arrowheads indicate basal c-MYC +ve sebocytes. (I) Asterisks indicate autofluorescent hair shaft.

(N) Schematic of SG showing undifferentiated sebocytes and early, mid, and late stages of terminal differentiation. Circular arrow indicates proliferating cells. HF, hair follicle; SD, sebaceous duct; PC, progenitor cell.

Scale bars, 40 μm. See also Figure S1.

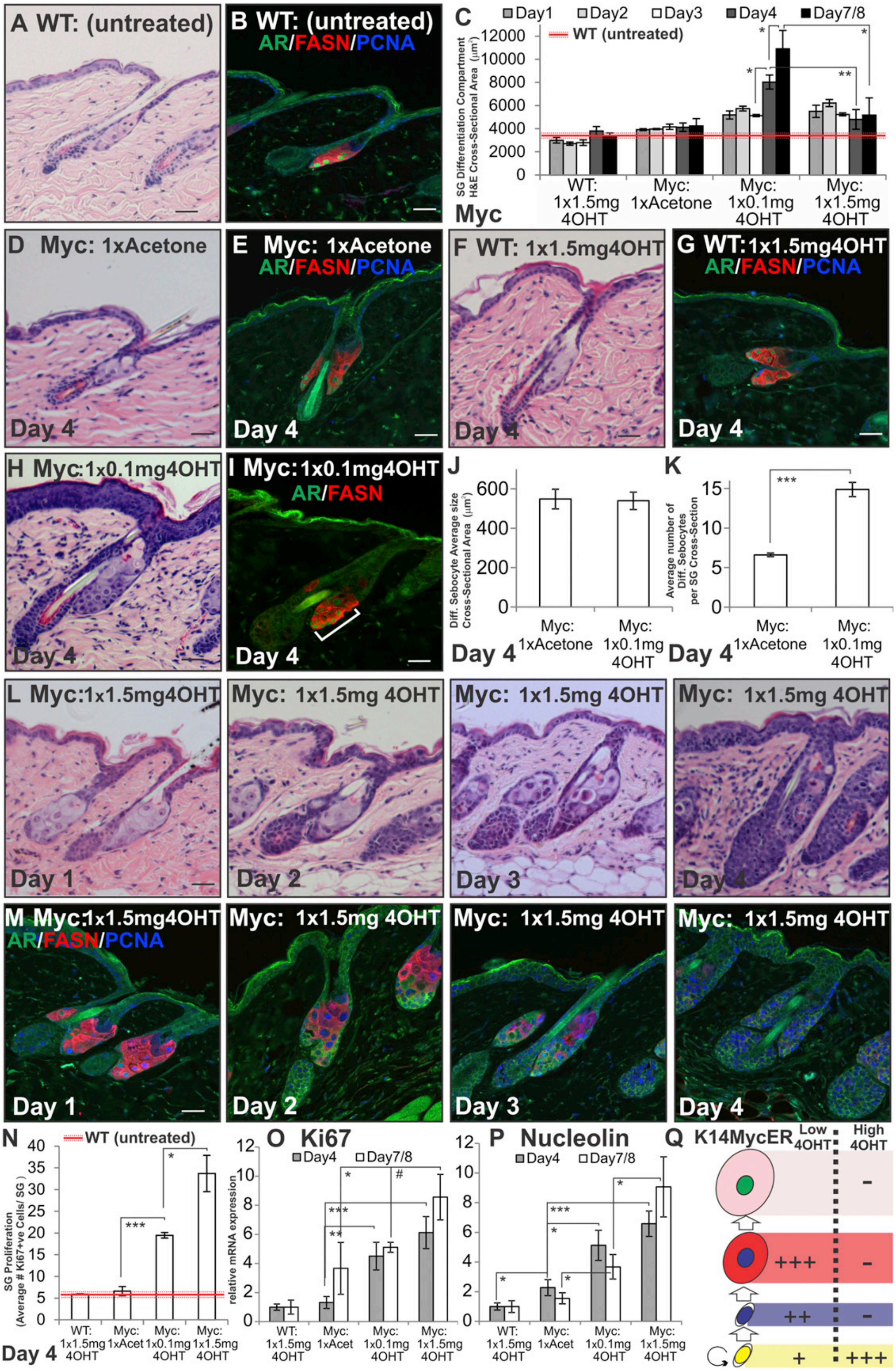

(legend on next page)

(Figure 2C; Figures S4A and S4C), exhibiting weakened expression of FASN (Figures S4B and S4D) and increased proliferation (Figures S4F and S4H).

In contrast to the effect of a low dose of 4OHT, by 4 days of high-dose 4OHT treatment, the SGs of *K14MycER* mice were filled with undifferentiated, immature sebocytes that frequently lacked nuclear AR and exhibited reduced FASN expression, indicating a collapse in AR signaling (Figures 2L and 2M). In some cases cytoplasmic AR was also reduced (see Figure 6C). This correlated with proliferative expansion of undifferentiated sebocytes at the base of the gland (Figures 2L and 2M). The reduction in the differentiation compartment (Figure 2C) was also observed 8 days after 4OHT treatment, the longest period for which mice could be monitored (Berta et al., 2010). MYC activity remained elevated at 4 and 7–8 days after one dose of 4OHT, as assessed by qRT-PCR of *Nucleolin* (Figure 2P), an established MYC target gene (Berta et al., 2010). In the IFE, formation of the cornified envelope persisted (Figure 2L), indicating the negative effects of high-dose MYC activation on differentiation were most pronounced in the SG lineage.

Although MYC stimulates AR activity and *AR* expression in prostate (Nadiminty et al., 2012), *Ar* mRNA levels in the skin of *K14MycER* mice were not affected by low or high doses of 4OHT (Figures S2D, S2E, S2F, and S2G). In AR-luciferase assays in immortalized human sebocytes, MYC repressed AR activity in the presence of testosterone but stimulated activity in cells treated with testosterone and the anti-androgen Casodex (Figure S2H), demonstrating MYC can regulate AR activity in a context-dependent manner.

These data suggest that in response to a low dose of 4OHT there is an accumulation of AR-positive differentiated sebocytes, whereas the impairment in sebaceous differentiation upon high-dose 4OHT treatment may reflect the inability of sebocytes to exit the proliferative basal cell compartment and acquire AR activity (Figure 2Q).

Effect of AR Activity on MYC-Induced SG Differentiation

To test the role of the AR in MYC-induced sebocyte differentiation, *K14MycER* mice were crossed onto the *AR-Shah* x *AR-TFM* strain background. Mice inheriting the *AR-Shah* reporter allele have functional ARs (Shah et al., 2004). Mice inheriting the *AR-TFM* mutation lack functional ARs (Gaspar et al., 1991). Their SGs are morphologically normal (Figure 3A), although sebum production was not assessed (Imperato-McGinley et al., 1993). *K14MycER AR-Shah* mice exhibited a similar response to low and high doses of 4OHT as the original *K14MycER* mice, although the SGs of control *K14MycER AR-Shah/AR-TFM* mice were slightly larger than original control *K14MycER* mice.

Compared to *K14MycER AR-Shah* mice, *K14MycER AR-TFM* mice showed a less pronounced induction of differentiation upon low-dose 4OHT treatment (Figures 3A–3C). FASN expression was reduced (Figure 3D), and there were increased numbers of Ki67+ve cells within the SG (Figures 3E and 3F). Similar results were observed when AR signaling in *K14MycER* mice was inhibited with high doses of Casodex (Figures S3I–S3K). As predicted from the block in differentiation, loss of AR did not affect the response to high-dose 4OHT (Figures 3A–3C). Although AR signaling is reported to regulate MYC protein stability (Bernard et al., 2003), we did not observe a significant change in *Nucleolin* mRNA expression (Figure 3G). We conclude that AR activity is required for efficient MYC-induced SG differentiation (Figure 3H). In contrast, genetic ablation of the *Ar* did not alter the IFE response to MYC or MYC-induced changes in telogen HFs (Arnold and Watt, 2001).

We next examined the effect of activating AR signaling with testosterone. Daily application of 2 mg testosterone had little effect on the number of differentiated sebocytes in WT 4OHT-treated and acetone-treated *K14MycER* mice of either gender, confirming that testosterone did not act directly on the MYCER fusion protein (Figure 4A). Testosterone did not enhance SG differentiation in *K14MycER* mice treated with a low dose of 4OHT for 4 days, suggesting that AR activity was already maximal in this condition (Figures S4G and S4H), although at 8 days SG hyperplasia was reduced (Figures S4E–S4H). In contrast, daily application of 2 mg testosterone to high-dose-treated *K14MycER* mice markedly stimulated differentiation and increased the SG differentiation compartment to the same extent as low-dose 4OHT (Figures 4A and 4D). Relocation of the AR to the nucleus and increased FASN expression confirmed testosterone restored AR activity (Figure 4B). Most sebocytes in skin treated with high-dose 4OHT and testosterone were AR and FASN+ve (Figures 4A and 4B), suggesting downregulation of AR activity might be required for later stages of maturation (Figure 1N). This was confirmed by examining the effect of Casodex on *K14MycER* mice treated with a low dose of 4OHT (Figures S3A–S3H).

The AR-dependent action of testosterone was confirmed by the competitive effect on SG differentiation of high doses of Casodex (Figure 4G) and by the inability of testosterone to rescue sebocyte differentiation in high-dose 4OHT *K14MycER AR-TFM* mice (Figure 4H). Testosterone increased levels of *Nucleolin* mRNA slightly, but the effect was not statistically significant (Figure 4F).

Figure 2. Effects of MYC Activation on SG Proliferation and Differentiation

(A–Q) WT and *K14MycER (Myc:)* mouse telogen back skin was untreated, acetone-treated (vehicle), or 4OHT-treated, as indicated. (A, D, F, H, and L) Skin sections were stained with H&E. (B, E, G, I, and M) Skin sections were stained with the antibodies shown. White bracket indicates expansion of AR-expressing sebocytes in (I). (C) Quantitation of SG differentiation compartment (average cross-sectional area occupied by differentiated sebocytes per SG) for the experiment described above. Red line represents average untreated WT measurement, and dashed red lines represent SEM. (J) Quantification of average size of individual differentiated sebocytes (cross-sectional area). (K) Average number of differentiated sebocytes per SG cross-section. (N) Quantitation of Ki67+ve sebocytes per SG. Red line represents untreated WT measurement. (O and P) qRT-PCR of *Ki67* and *Nucleolin* mRNA levels relative to *Gapdh*. (Q) Schematic summary of changes relative to WT SG of treating *K14MycER* mice with low or high dose 4OHT. See Figure 1N for stages in sebocytes differentiation. Increases in cell number are represented by +, ++, or +++, according to the strength of the effect. Reduction in cell number is represented by −.

Three to nine mice were examined per condition. Error bars represent SEM #$p < 0.06$, *$p < 0.05$, **$p < 0.01$, and ***$p < 0.005$. Scale bars 40 μm. See also Figure S2.

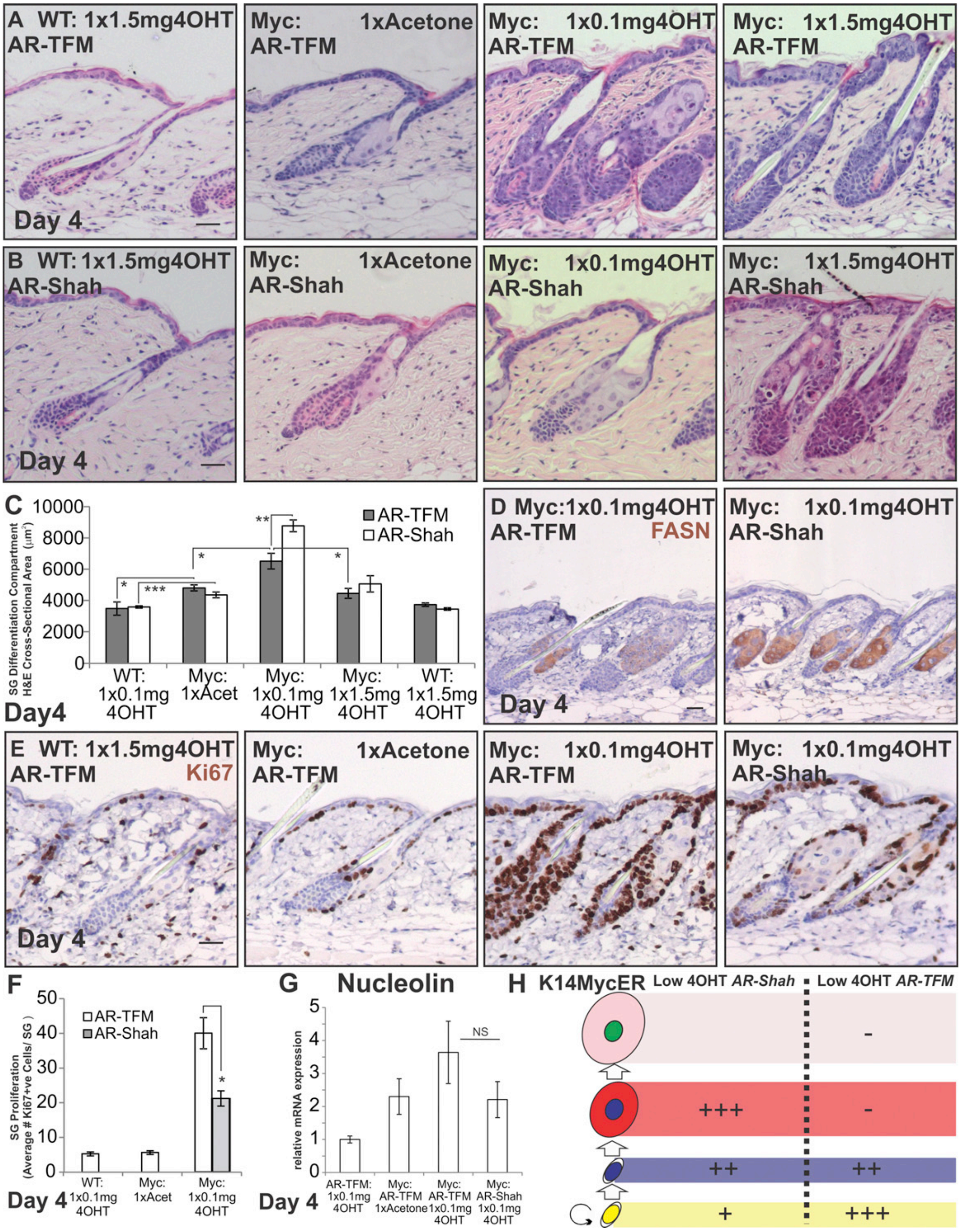

Figure 3. Effect of Loss of AR Function during MYC Activation

(A–H) WT and *K14MycER* mice *(Myc:)* were crossed with *AR-Shah* or *AR-TFM* mice and treated as indicated. (A and B) Skin sections were stained with H&E. (C) Quantitation of size of SG differentiation compartment. (D and E) Immunostaining (brown) for antibodies indicated. (F) Quantitation of Ki67+ve sebocytes. (G) qRT-PCR of *Nucleolin* mRNA levels relative to *Gapdh*. (H) Schematic summary of the effects of AR loss of function. See Figure 1N for stages in sebocyte differentiation. Increases in cell number are represented by +, ++, or +++, according to the strength of the effect. Reduction in cell number is represented by −.
Three to seven mice were examined per condition. Error bars represent SEM. *p < 0.05, **p < 0.01, and ***p < 0.005. Scale bars, 40 µm. See also Figure S3.

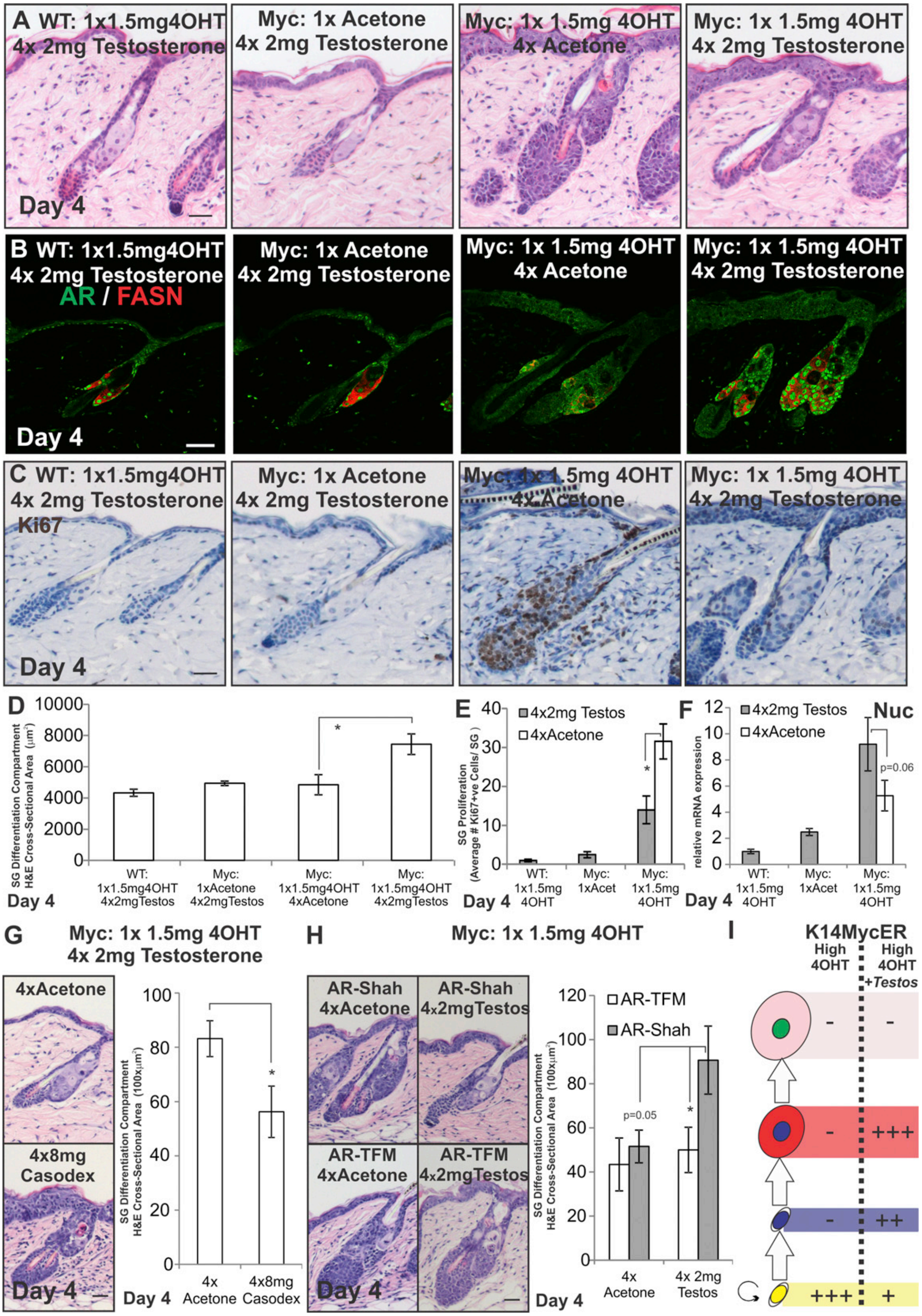

(legend on next page)

The differentiation promoting effects of testosterone in *K14MycER* mice treated with a high dose of 4OHT are summarized in Figure 4I.

Role of p53 in MYC-Induced Sebocyte Differentiation

Oncogenic levels of MYC can activate p53 via DNA damage (Hong et al., 2006; Murphy et al., 2008; Pusapati et al., 2006). p53 can inhibit *AR* gene expression (Alimirah et al., 2007) and activity (Shenk et al., 2001) and block lipogenesis (Hallenborg et al., 2009). We therefore investigated whether p53 modulated the *K14MycER* sebaceous gland phenotype.

MYC activation induced DNA damage throughout the epidermis, as detected by γ-H2AX expression (Pusapati et al., 2006) (Figure 5A). When MYC was activated with high-dose 4OHT, accumulation of active p53 was observed with an antibody to nuclear p53 (Figures 5B and 5G). Antibody specificity was confirmed on *p53null* mice (Figure S5A). However, the number of cells expressing the apoptotic marker cleaved caspase-3 was very low even when high levels of MYC were induced (Figure 5C). The induction of *p53* was confirmed by qRT-PCR (Figure 5D); in contrast, levels of total *p63* and *p73* were not significantly changed (Figures 5E and 5F). Given that *p53* was induced within 4 days of MYC activation, it is likely to be wild-type and not mutant.

When *K14MycER* mice were treated with 4OHT and testosterone, nuclear p53 protein (but not mRNA) levels were reduced throughout the epidermis (Figures 5H–5J). p53 activity was higher in *K14MycER AR-TFM* mice treated with a low dose of 4OHT than in *K14MycER AR-Shah* mice (Figures 5K and 5L), confirming that AR functions to repress p53 activity (Nantermet et al., 2004).

To examine whether p53 activation in *K14MycER* mice treated with a high dose of 4OHT contributed to the inhibition of SG differentiation, mice were crossed onto a *p53null* background. In the SG, the effect was most profound and restored differentiation, with increased numbers of cells expressing nuclear AR and FASN, relative to mice that were heterozygous for *p53* (Figures 6A–6C). IVL showed some restoration in expression, but BLIMP1 expression was not restored (Figure S5B). Genetic ablation of *p53* partially reduced MYC-induced IFE hyperproliferative changes but did not alter MYC-induced changes in telogen HFs (data not shown).

p53 deletion did not increase *Ar* (Figure 6D) or *Nucleolin* mRNA expression (Figure 6E) but increased MYC-dependent induction of *Fasn* and *Pparγ* (Figures 6F and 6G). Consistent with the stimulation of differentiation, there was reduced expression of *Ki67* (Figure 6H) and of *Keratin 7* (K7), a sebaceous lineage marker (Zouboulis et al., 1999) that is expressed in the SG basal layer (Ju et al., 2011) (Figure 6I). Conversely, when p53 activation in 4OHT-treated *K14MycER* mice was enhanced by daily application of camptothecin (a topoisomerase inhibitor that causes DNA breaks and activates p53 [Rudolf et al., 2011]) (Figures S5C–S5F), AR activity and proliferation in the SG lineage were reduced resulting in a reduction in gland size (Figure S5F). Testosterone coapplication partially antagonized the effects of camptothecin on the SG (Figures S5C–S5F).

We were able to generate a single *K14MycER p53null AR-TFM* mouse. This mouse exhibited reduced SG differentiation and enhanced proliferation compared to *K14MycER p53null* controls, with proliferation persisting in FASN+ve sebocytes. Sebocytes were also detected in the IFE (Figure 6J, see arrowhead).

Collectively, these experiments suggest that p53 activation resulting from DNA damage induced by high levels of MYC, contributes to the inhibition of SG differentiation and disruption of AR signaling (Figure 6K). Therefore, AR and p53 form an axis of mutual antagonism controlling the outcome of MYC activation in the SG. For further details of all prior results, please refer to the Extended Results.

AR and p53 Expression in Human Sebaceous Tumors

To place our observations into the context of human patho-physiology, we examined expression of MYC, Ki67, AR, and p53 in human sebaceous tumors. Human SGs differ from mouse back skin SGs because they are larger and have a multilobed structure. In this regard the human SG resembles the murine preputial gland (Figures 7A and 7B). In both human scalp SGs and mouse preputial glands, we observed significant overlap in the expression of endogenous MYC and the AR, as well as MYC and the proliferative Ki67 basal compartment. We also observed active p53 in basal cells at low frequency (Figures 7A and 7B).

Differentiation status in tumors was assessed in H&E-stained sections and correlated inversely with the number of Ki67+ve cells, with one exception (Figure 7B). MYC was present in all tumors but often at very low levels, and the level of MYC expression exhibited no correlation with proliferation, differentiation status, or tumor type (Figure 7B).

Although p53 is often mutated in sebaceous carcinoma (Kiyosaki et al., 2010), many of these transcriptionally inactive mutations can inhibit AR activity (Nesslinger et al., 2003). Tumors exhibiting reduced differentiation had a higher proportion of p53+ve sebocytes compared to AR+ve sebocytes, whereas those with increased differentiation had an equal or higher proportion of AR+ve sebocytes compared to p53+ve sebocytes. Three specimens exhibited multiple neoplasms within a single section, with distinct regions of adenoma (A) and carcinoma (C). These confirmed that the differences between high and low differentiation status tumors were not due to patient-specific

Figure 4. Testosterone Modulation of AR Activity during MYC Activation

(A–I) WT, *K14MycER* (Myc:), *K14MycER AR-Shah*, and *K14MycER AR-TFM* mice were treated as indicated. (A) Skin sections were stained with H&E. (B and C) Skin sections were stained with the antibodies shown. (D) Quantitation of the SG differentiation compartment. (E) Quantitation of Ki67+ve sebocytes per SG. (F) qRT-PCR of *Nucleolin* (Nuc) mRNA levels relative to *Gapdh*. (G and H) Skin sections were stained with H&E. Quantitation of SG differentiation compartment size is shown. (I) Schematic summary of the effect of testosterone on *K14MycER* mice treated with high-dose 4OHT. See Figure 1N for stages in sebocytes differentiation. Increases in cell number are represented by +, ++, or +++, according to the strength of the effect. Reduction in cell number is represented by −.

Three to six mice were examined per condition. Error bars represent SEM. *$p < 0.05$, **$p < 0.01$, and ***$p < 0.005$. Scale bars, 40 μm. See also Figures S3 and S4.

Cell
PRESS
Open
ACCESS

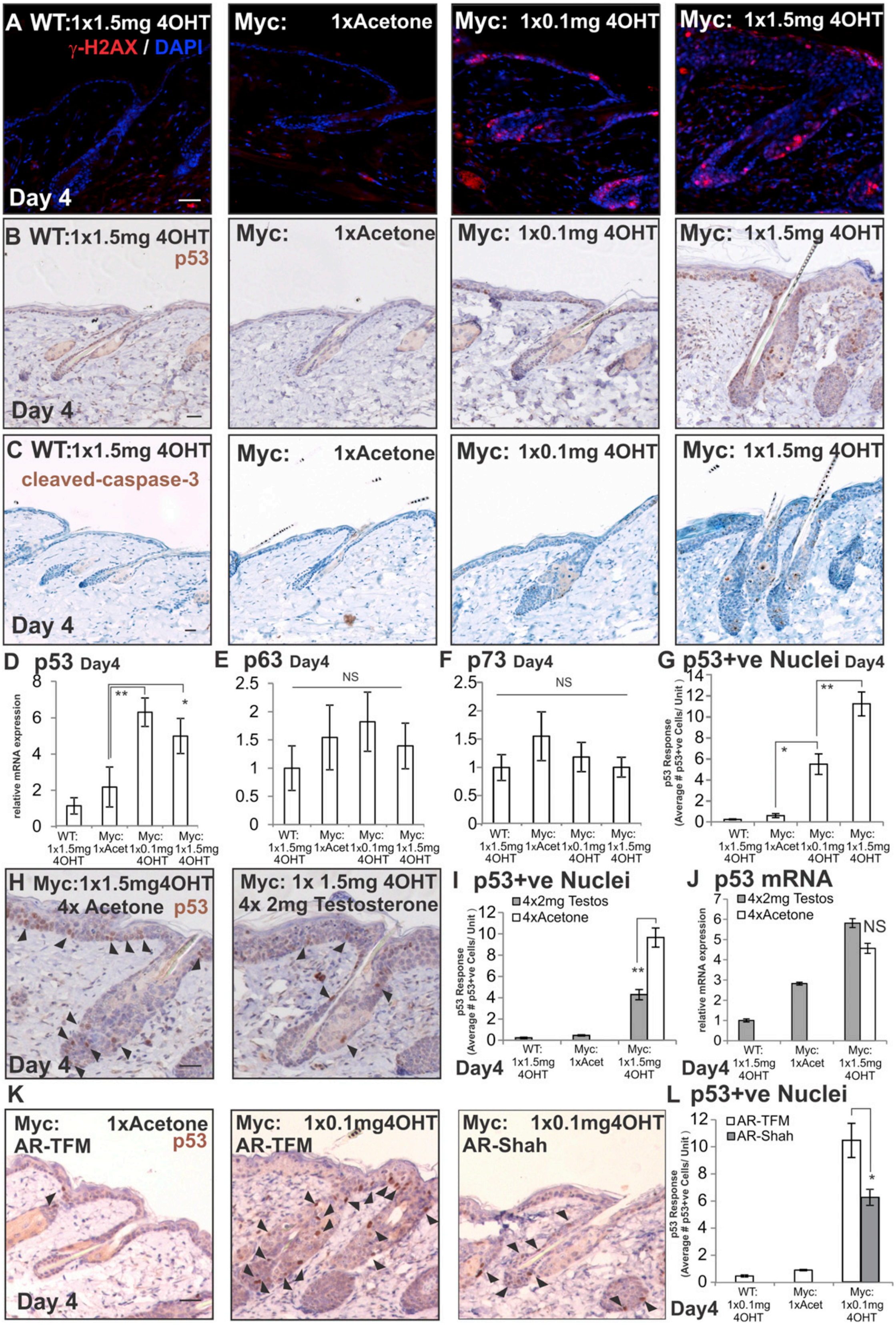

(legend on next page)

effects (Figures 7B and 7C). Our results suggest that mutual antagonism of AR and p53 may contribute to progression of sebaceous carcinoma.

DISCUSSION

Our findings support a model whereby when MYC is overactivated with a low dose of 4OHT, the AR inhibits proliferation and stimulates the onset of differentiation. However, greater MYC activity induces a p53 response as a result of DNA-damage, consistent with previous observations (Pusapati et al., 2006). The p53 response (increased nuclear p53 protein and its downstream consequences) inhibits AR activity and without the AR to trigger the onset of differentiation, cells continue proliferating in response to MYC and accumulate as undifferentiated sebocytes. The nonapoptotic p53 response could reflect the reported ability of MYC to inhibit p53-mediated apoptosis (Ceballos et al., 2000).

The AR can promote or retard sebocyte proliferation, depending on androgen type and the body site from which sebocytes are derived (Akamatsu et al., 1992). The phenotype of *AR-TFM* mice highlights the antiproliferative role of the AR in murine back skin following MYC activation but shows that the AR is not required for SG differentiation during normal skin homeostasis (Bayer-Garner et al., 1999; Rosenfield et al., 1998). In rodent meibomian sebocytes, the AR functions early in differentiation to initiate lipogenesis and lipid metabolism via transcriptional regulation of genes, such as *Fasn* and *Ppar*δ (Schirra et al., 2005, 2006). Placing the AR upstream of the lipogenic program is consistent with our observations because peak AR expression was observed just before the onset of differentiation and then persisted during the first accumulation of lipids, marked by FASN and PPARγ. Downregulation of AR activity late in sebocyte differentiation may play a role in the final stages of sebocyte maturation because low doses of the anti-androgen Casodex facilitated lipid accumulation (Figure S3D). The AR also appears to have a role in repressing p53 activation (Figures 5H, 5I, 5K, and 5L; Figures S4I and S4J and S5C–S5G), as previously reported in prostate (Nantermet et al., 2004).

Loss of p53, like loss of AR, did not affect SG homeostasis, that is, the normal balance between proliferation and differentiation, although p53 is expressed in undifferentiated human and mouse sebocytes (Figures 7A and 7B). p53 deletion restored SG differentiation in high-dose 4OHT-treated *K14MycER* mice, and this rescue was diminished in the absence of AR signaling, highlighting the antagonistic effects of AR and p53. Additional MYC-induced differentiation factors may be inhibited by p53, such as PPARγ (Figure 6G), which can bypass the requirement for androgens in normal SG differentiation (Rosenfield et al., 1999). Although p53 activation in *K14MycER* mice did not induce apoptosis, epidermal *Setd8* deletion triggers apoptotic p53 activation, resulting in loss of the IFE and SGs (Driskell et al., 2011a). Increasing p53 activity with camptothecin (Figures S5E and S5G) resulted in an apoptotic phenotype that was similar to the early stages of epidermal *Setd8* deletion (Driskell et al., 2011a), demonstrating that different thresholds of p53 activation can have distinct outcomes.

Our observation that AR and p53 are mutually antagonistic in regulating MYC-induced sebocyte differentiation is relevant to human sebaceous carcinoma. As in the mouse model, increased numbers of p53-positive sebocytes correlated fewer AR-positive sebocytes, reduced differentiation, and poor prognosis in the tumors (Figure 7) (Bayer-Garner et al., 1999; Hasebe et al., 1994; Hayashi et al., 1994; Izumi et al., 2008). MYC was detected in all the tumors we examined and did not correlate with Ki67 expression, consistent with a role of MYC in regulating both proliferation and differentiation. Although in contrast to *K14MycER* mice, p53 is often mutated in sebaceous carcinoma (Kiyosaki et al., 2010) as a result of DNA damage, and these mutations are still capable of inhibiting AR activity when tested in prostate cells (Nesslinger et al., 2003). It is possible therefore that within p53-positive sebaceous carcinomas, p53 accumulation impairs AR signaling without inhibiting proliferation (Figures 5, 6, and 7). p53 could interfere posttranslationally with AR activity by disrupting AR homodimerization and DNA binding (Shenk et al., 2001). Alternatively, because AR and p53 protein stability are both regulated by murine double minute 2 (MDM2), AR downregulation in tumors could be due to activation of MDM2 in response to increased p53 (Kulikov et al., 2010; Lin et al., 2002). Finally, p53 could indirectly affect AR signaling by reducing local androgen synthesis in the skin (Chen et al., 2006, 2010; Hallenborg et al., 2009). The observation that p53 deletion confers resistance to spontaneous and chemically induced epidermal papillomas and squamous cell carcinomas (Greenhalgh et al., 1996) suggests that p53 activation also influences differentiation in the interfollicular epidermis.

In conclusion, our study identifies an AR/p53 axis that determines the outcome of MYC overactivation in the SG. When MYC activity is moderately elevated, the AR functions to prevent p53 activation, terminate proliferation, and promote the onset of differentiation. However, in response to high MYC activity, p53 can block AR signaling and thereby inhibit differentiation, leading to expansion of undifferentiated sebocytes. Our observations help to explain how MYC, an oncogene, can trigger SG differentiation and how activation of p53 can facilitate proliferation of undifferentiated cells in human sebaceous carcinomas via downregulation of the AR.

EXPERIMENTAL PROCEDURES

Transgenic Mice

K14MycER transgenic mouse founder line 2184C.1 (Arnold and Watt, 2001) was used for this study and maintained on a C57/Bl6 x CBA F1 background.

Figure 5. Effect of MYC and AR Activation on p53 Expression, DNA Double Strand Breaks, and Apoptosis

(A–L) WT and *K14MycER* (Myc:) mice were treated as indicated. (A) Skin sections were labeled with antibodies to γ-H2AX with DAPI nuclear counterstain. (B, H, and K) Skin sections were labeled with antibodies to p53 (arrowheads indicate positive nuclei). (C) Skin sections were labeled with antibodies to cleaved caspase 3. (D–F) qRT-PCR of mRNA levels relative to *Gapdh*. (G, I, and L) Quantitation of p53+ve nuclei per unit area of skin (HF, SG, and overlying IFE). (J) qRT-PCR of *p53* mRNA levels relative to *Gapdh*.

Three to nine mice were examined per condition. Error bars represent SEM. *p < 0.05, **p < 0.01. Scale bars, 40 μm.

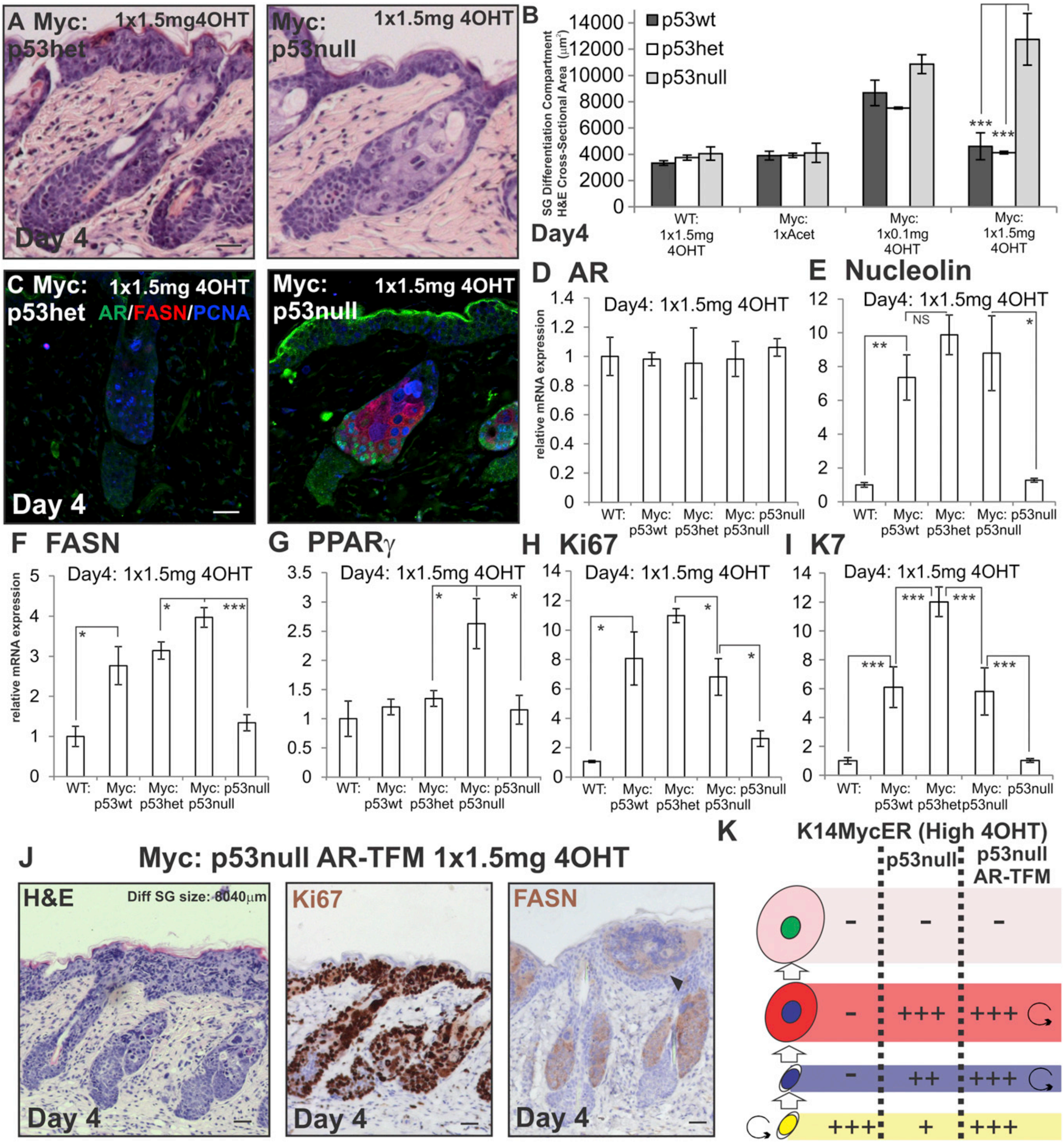

Figure 6. Loss of p53 Function during MYC Activation

(A–K) Mice were treated as indicated. (A and J) Skin sections were stained with H&E. (B) Quantitation of SG differentiation compartment. (C and J) Skin sections were stained with the antibodies shown. Arrowhead in (J) indicates sebocyte differentiation within the IFE. (D–I) qRT-PCR of mRNA levels relative to *Gapdh*. (K) Schematic summary of effects of loss of p53 and combined loss of p53 and the AR. See Figure 1N for stages in sebocytes differentiation. Increases in cell number are represented by +, ++, or +++, according to the strength of the effect. Reduction in cell number is represented by −.

Scale bars, 40 μm. Error bars represent SEM. Three to five mice were examined per condition, except n = 1 for triple cross. NS, not significant. *$p < 0.05$, **$p < 0.01$, ***$p < 0.005$. See also Figure S5.

A

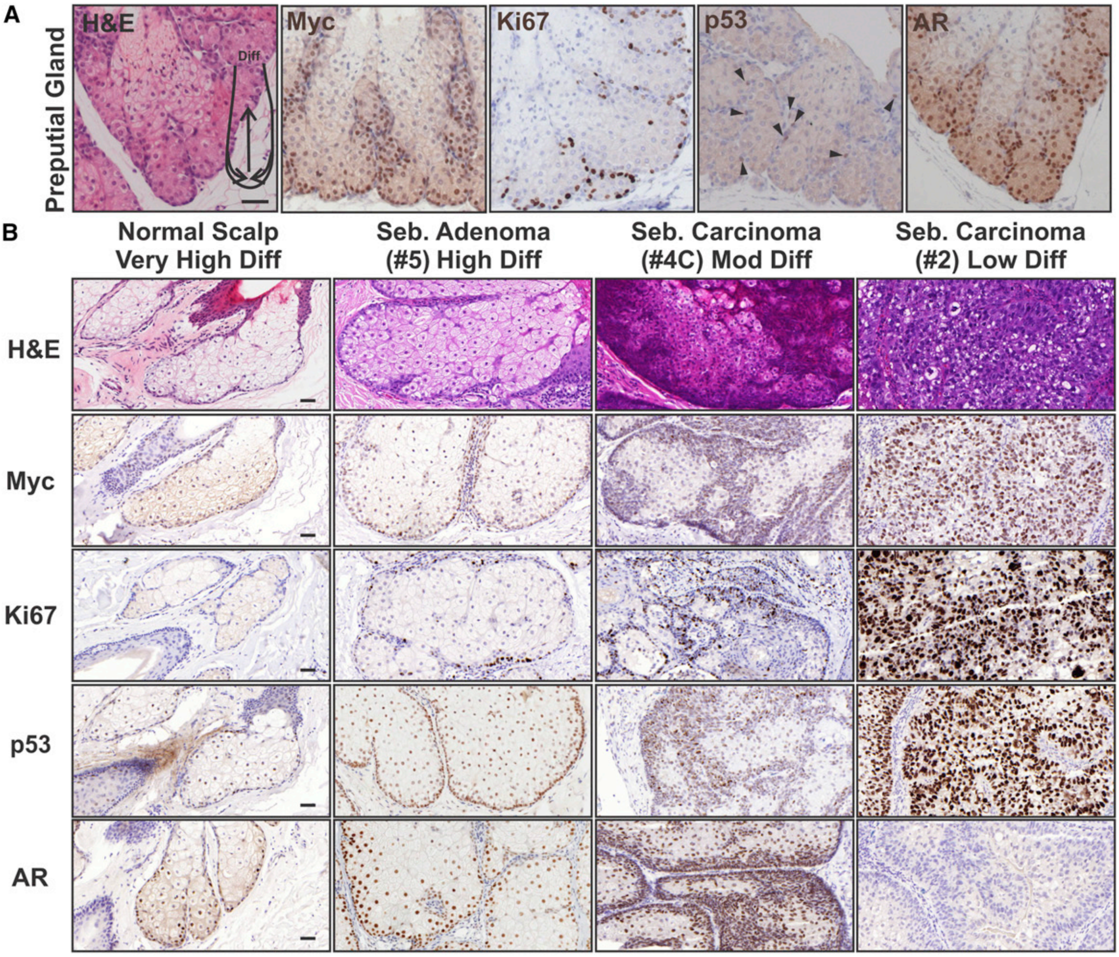

C Table of Human Patient Data:

Sample ID	Classification	Myc	Ki67	p53	AR	Diff
Scalp	Normal	++	-	+	+	Very High
#7A	Sebaceous Adenoma	+	+	+	+	High
#8A	Sebaceous Adenoma	+	+	-/+	+	High
#4A	Sebaceous Adenoma	++	+	+	+	High
#5	Sebaceous Adenoma	++	+	++	++	High
#6	Sebaceous Adenoma	+	++	++	+++	High
#4C	Sebaceous Carcinoma	+++	++	++	++++	Moderate
#3	Sebaceous Carcinoma	++	+++	++	+++	Moderate
#7C	Sebaceous Carcinoma	+	+++	+++	++	Low
#9	Sebaceous Carcinoma	+	+++	++++	++	Low
#1	Sebaceous Carcinoma	+++	+++	+++	-	Low
#11	Sebaceous Carcinoma	+	++++	+++	-	Low
#2	Sebaceous Carcinoma	++++	++++	++++	-	Low
#12	Sebaceous Carcinoma	++	++++	++++	-	Low
#10	Sebaceous Carcinoma	-/+	+++	++++	+	Very Low
#8C	Sebaceous Carcinoma	-/+	+	-/+	-	Very Low

Figure 7. p53 and AR Expression in Human Sebaceous Tumors

(A) Mouse preputial gland sections were stained with H&E or the antibodies shown. Diagram on H&E indicates differentiation within each sebaceous lobe. Arrowheads indicate p53+ve cells.

(B) Representative tumor sections are stained with H&E or the antibodies shown. Scale bars 40 μm.

(C) Summary of immunostaining and differentiation status of individual tumors. Staining was scored as follows. − negative, −/+ almost entirely negative, + to ++++ increasing proportion of positive cells, with ++++ indicating almost every cell positive.

The other strains of mice used in crosses are described in the Extended Experimental Procedures. Mice were treated once with 100 μl acetone or 0.1 or 1.5 mg 4OHT (Sigma-Aldrich H6278; Sigma-Aldrich, St. Louis, MO, USA) dissolved in 100 μl acetone. 4OHT was applied to clipped lower back skin, and mice were analyzed 1–8 days later. In some experiments, mice additionally received daily doses of 100 μl of acetone and/or 2 mg testosterone (Testos; Sigma-Aldrich T1500) and/or 2, 4, or 8 mg Casodex (bicalutamide antiandrogen; Sigma-Aldrich B9061) in 100 μl acetone. See Figure S2A for treatment illustration and the Extended Experimental Procedures.

All experiments were performed on a minimum of three mice per condition, with the exception of *K14MycER p53null AR-TFM* mice. Experiments were subject to Cancer Research UK ethical review and performed under the terms of a UK Government Home Office license.

Immunofluorescence, Immunohistochemistry, and Microscopy

Primary and secondary antibodies and labeling procedures are described in the Extended Experimental Procedures and Table S1. Immunofluorescence (IF) slides were counterstained with the nuclear dye DAPI. Immunohistochemistry slides were counterstained with hematoxylin.

qRT-PCR

RNA isolation, cDNA preparation, and qRT-PCR were performed using the Trizol method as described previously (Driskell et al., 2011a) and described in detail in the Extended Experimental Procedures.

Human Tissue

Samples were collected, diagnosed, and provided by H.G. and S.R.Q. or S.A. and K.N. All samples were obtained with informed consent and processed for research in accordance with the recommendations of the relevant local ethics committees: CRUK Cambridge Research Institute (number 08/H0306/30), German Medical Council, and/or the Japanese Ministry of Health, Labor, and Welfare.

Quantitation and Statistics

Quantitation of Ki67, p53+ve cells, and the average cross-sectional area of the SG differentiation compartment were determined from at least ten 10x images of H&E-stained tissue sections per mouse. Only vertical sections of back skin were quantitated. The SG differentiation compartment was identified in H&E sections on the basis of differentiating sebocytes exhibiting pale and enlarged cytoplasm from lipid accumulation. Examples of how measurements were made are shown in Figure S2B. Statistical analysis was performed using the unpaired Student's t test.

SUPPLEMENTAL INFORMATION

Supplemental Information includes Extended Results, Extended Experimental Procedures, five figures, and one table and can be found with this article online at http://dx.doi.org/10.1016/j.celrep.2013.01.013.

LICENSING INFORMATION

This is an open-access article distributed under the terms of the Creative Commons Attribution License, which permits unrestricted use, distribution, and reproduction in any medium, provided the original author and source are credited.

ACKNOWLEDGMENTS

We thank K. Jensen, R. Driskell, and M. Mastrogiannaki for advice; E. Nascimento and M. Frye for reagents; A. Lamb for mammalian Myc expression constructs; and CRI and SCI core staff for expert technical assistance, in particular P. Humphreys, M. McLeish, H. Skelton, A. Mowbray, M. Webb, and J. Fox. F.M.W. gratefully acknowledges the financial support of the MRC, Wellcome Trust, CRUK, and the EU FP7 programme. We also acknowledge the support of the University of Cambridge and Hutchison Whampoa Ltd. K.K. is the recipient of a MRC PhD studentship. P.J.S. is the recipient of a CRUK PhD studentship.

Received: March 27, 2012
Revised: October 28, 2012
Accepted: January 14, 2013
Published: February 7, 2013

REFERENCES

Akamatsu, H., Zouboulis, C.C., and Orfanos, C.E. (1992). Control of human sebocyte proliferation in vitro by testosterone and 5-alpha-dihydrotestosterone is dependent on the localization of the sebaceous glands. J. Invest. Dermatol. *99*, 509–511.

Alimirah, F., Panchanathan, R., Chen, J., Zhang, X., Ho, S.M., and Choubey, D. (2007). Expression of androgen receptor is negatively regulated by p53. Neoplasia *9*, 1152–1159.

Arnold, I., and Watt, F.M. (2001). c-Myc activation in transgenic mouse epidermis results in mobilization of stem cells and differentiation of their progeny. Curr. Biol. *11*, 558–568.

Azzi, L., El-Alfy, M., and Labrie, F. (2006). Gender differences and effects of sex steroids and dehydroepiandrosterone on androgen and oestrogen alpha receptors in mouse sebaceous glands. Br. J. Dermatol. *154*, 21–27.

Bayer-Garner, I.B., Givens, V., and Smoller, B. (1999). Immunohistochemical staining for androgen receptors: a sensitive marker of sebaceous differentiation. Am. J. Dermatopathol. *21*, 426–431.

Bernard, D., Pourtier-Manzanedo, A., Gil, J., and Beach, D.H. (2003). Myc confers androgen-independent prostate cancer cell growth. J. Clin. Invest. *112*, 1724–1731.

Berta, M.A., Baker, C.M., Cottle, D.L., and Watt, F.M. (2010). Dose and context dependent effects of Myc on epidermal stem cell proliferation and differentiation. EMBO Mol. Med. *2*, 16–25.

Buitrago, W., and Joseph, A.K. (2008). Sebaceous carcinoma: the great masquerader: emgerging concepts in diagnosis and treatment. Dermatol. Ther. *21*, 459–466.

Ceballos, E., Delgado, M.D., Gutierrez, P., Richard, C., Müller, D., Eilers, M., Ehinger, M., Gullberg, U., and León, J. (2000). c-Myc antagonizes the effect of p53 on apoptosis and p21WAF1 transactivation in K562 leukemia cells. Oncogene *19*, 2194–2204.

Chang, D.H., Cattoretti, G., and Calame, K.L. (2002). The dynamic expression pattern of B lymphocyte induced maturation protein-1 (Blimp-1) during mouse embryonic development. Mech. Dev. *117*, 305–309.

Chen, W., Tsai, S.J., Liao, C.Y., Tsai, R.Y., Chen, Y.J., Pan, B.J., Hung, C.L., and Zouboulis, C.C. (2006). Higher levels of steroidogenic acute regulatory protein and type I 3beta-hydroxysteroid dehydrogenase in the scalp of men with androgenetic alopecia. J. Invest. Dermatol. *126*, 2332–2335.

Chen, W., Tsai, S.J., Sheu, H.M., Tsai, J.C., and Zouboulis, C.C. (2010). Testosterone synthesized in cultured human SZ95 sebocytes derives mainly from dehydroepiandrosterone. Exp. Dermatol. *19*, 470–472.

Cui, C.Y., Durmowicz, M., Ottolenghi, C., Hashimoto, T., Griggs, B., Srivastava, A.K., and Schlessinger, D. (2003). Inducible mEDA-A1 transgene mediates sebaceous gland hyperplasia and differential formation of two types of mouse hair follicles. Hum. Mol. Genet. *12*, 2931–2940.

Deplewski, D., and Rosenfield, R.L. (1999). Growth hormone and insulin-like growth factors have different effects on sebaceous cell growth and differentiation. Endocrinology *140*, 4089–4094.

Driskell, I., Oda, H., Blanco, S., Nascimento, E., Humphreys, P., and Frye, M. (2011a). The histone methyltransferase Setd8 acts in concert with c-Myc and is required to maintain skin. EMBO J. *31*, 616–629.

Gaspar, M.L., Meo, T., Bourgarel, P., Guenet, J.L., and Tosi, M. (1991). A single base deletion in the Tfm androgen receptor gene creates a short-lived messenger RNA that directs internal translation initiation. Proc. Natl. Acad. Sci. USA *88*, 8606–8610.

Greenhalgh, D.A., Wang, X.J., Donehower, L.A., and Roop, D.R. (1996). Paradoxical tumor inhibitory effect of p53 loss in transgenic mice expressing epidermal-targeted v-rasHa, v-fos, or human transforming growth factor alpha. Cancer Res. *56*, 4413–4423.

Hallenborg, P., Feddersen, S., Madsen, L., and Kristiansen, K. (2009). The tumor suppressors pRB and p53 as regulators of adipocyte differentiation and function. Expert Opin. Ther. Targets *13*, 235–246.

Hasebe, T., Mukai, K., Yamaguchi, N., Ishihara, K., Kaneko, A., Takasaki, Y., and Shimosato, Y. (1994). Prognostic value of immunohistochemical staining for proliferating cell nuclear antigen, p53, and c-erbB-2 in sebaceous gland carcinoma and sweat gland carcinoma: comparison with histopathological parameter. Mod. Pathol. *7*, 37–43.

Hayashi, N., Furihata, M., Ohtsuki, Y., and Ueno, H. (1994). Search for accumulation of p53 protein and detection of human papillomavirus genomes in sebaceous gland carcinoma of the eyelid. Virchows Arch. *424*, 503–509.

Hayward, W.S., Neel, B.G., and Astrin, S.M. (1981). Activation of a cellular onc gene by promoter insertion in ALV-induced lymphoid leukosis. Nature *290*, 475–480.

Honeycutt, K.A., and Roop, D.R. (2004). c-Myc and epidermal stem cell fate determination. J. Dermatol. *31*, 368–375.

Hong, S., Pusapati, R.V., Powers, J.T., and Johnson, D.G. (2006). Oncogenes and the DNA damage response: Myc and E2F1 engage the ATM signaling pathway to activate p53 and induce apoptosis. Cell Cycle *5*, 801–803.

Horsley, V., O'Carroll, D., Tooze, R., Ohinata, Y., Saitou, M., Obukhanych, T., Nussenzweig, M., Tarakhovsky, A., and Fuchs, E. (2006). Blimp1 defines a progenitor population that governs cellular input to the sebaceous gland. Cell *126*, 597–609.

Imperato-McGinley, J., Gautier, T., Cai, L.Q., Yee, B., Epstein, J., and Pochi, P. (1993). The androgen control of sebum production. Studies of subjects with dihydrotestosterone deficiency and complete androgen insensitivity. J. Clin. Endocrinol. Metab. *76*, 524–528.

Izumi, M., Mukai, K., Nagai, T., Matsubayashi, J., Iwaya, K., Chiu, C.S., and Goto, H. (2008). Sebaceous carcinoma of the eyelids: thirty cases from Japan. Pathol. Int. *58*, 483–488.

Jensen, K.B., Collins, C.A., Nascimento, E., Tan, D.W., Frye, M., Itami, S., and Watt, F.M. (2009). Lrig1 expression defines a distinct multipotent stem cell population in mammalian epidermis. Cell Stem Cell *4*, 427–439.

Ju, Q., Fimmel, S., Hinz, N., Stahlmann, R., Xia, L., and Zouboulis, C.C. (2011). 2,3,7,8-Tetrachlorodibenzo-p-dioxin alters sebaceous gland cell differentiation in vitro. Exp. Dermatol. *20*, 320–325.

Kiyosaki, K., Nakada, C., Hijiya, N., Tsukamoto, Y., Matsuura, K., Nakatsuka, K., Daa, T., Yokoyama, S., Imaizumi, M., and Moriyama, M. (2010). Analysis of p53 mutations and the expression of p53 and p21WAF1/CIP1 protein in 15 cases of sebaceous carcinoma of the eyelid. Invest. Ophthalmol. Vis. Sci. *51*, 7–11.

Kulikov, R., Letienne, J., Kaur, M., Grossman, S.R., Arts, J., and Blattner, C. (2010). Mdm2 facilitates the association of p53 with the proteasome. Proc. Natl. Acad. Sci. USA *107*, 10038–10043.

Latham, J.A., Redfern, C.P., Thody, A.J., and De Kretser, T.A. (1989). Immunohistochemical markers of human sebaceous gland differentiation. J. Histochem, ed. *37*, 729–734.

Laurent, S.J., Mednieks, M.I., and Rosenfield, R.L. (1992). Growth of sebaceous cells in monolayer culture. In Vitro Cell. Dev. Biol. *28A*, 83–89.

Lin, H.K., Wang, L., Hu, Y.C., Altuwaijri, S., and Chang, C. (2002). Phosphorylation-dependent ubiquitylation and degradation of androgen receptor by Akt require Mdm2 E3 ligase. EMBO J. *21*, 4037–4048.

Lo Celso, C., Berta, M.A., Braun, K.M., Frye, M., Lyle, S., Zouboulis, C.C., and Watt, F.M. (2008). Characterization of bipotential epidermal progenitors derived from human sebaceous gland: contrasting roles of c-Myc and beta-catenin. Stem Cells *26*, 1241–1252.

Magnúsdóttir, E., Kalachikov, S., Mizukoshi, K., Savitsky, D., Ishida-Yamamoto, A., Panteleyev, A.A., and Calame, K. (2007). Epidermal terminal differentiation depends on B lymphocyte-induced maturation protein-1. Proc. Natl. Acad. Sci. USA *104*, 14988–14993.

Markova, M.S., Zeskand, J., McEntee, B., Rothstein, J., Jimenez, S.A., and Siracusa, L.D. (2004). A role for the androgen receptor in collagen content of the skin. J. Invest. Dermatol. *123*, 1052–1056.

McCulley, J.P., and Shine, W.E. (2004). The lipid layer of tears: dependent on meibomian gland function. Exp. Eye Res. *78*, 361–365.

Montagna, W., Ellis, R.A., and Silver, A.F. (1963). Advances in Biology of Skin. The Sebaceous Glands, *Volume 4* (Pergamon: Oxford).

Murphy, D.J., Junttila, M.R., Pouyet, L., Karnezis, A., Shchors, K., Bui, D.A., Brown-Swigart, L., Johnson, L., and Evan, G.I. (2008). Distinct thresholds govern Myc's biological output in vivo. Cancer Cell *14*, 447–457.

Nadiminty, N., Tummala, R., Lou, W., Zhu, Y., Zhang, J., Chen, X., eVere White, R.W., Kung, H.J., Evans, C.P., and Gao, A.C. (2012). MicroRNA let-7c suppresses androgen receptor expression and activity via regulation of Myc expression in prostate cancer cells. J. Biol. Chem. *287*, 1527–1537.

Nantermet, P.V., Xu, J., Yu, Y., Hodor, P., Holder, D., Adamski, S., Gentile, M.A., Kimmel, D.B., Harada, S., Gerhold, D., et al. (2004). Identification of genetic pathways activated by the androgen receptor during the induction of proliferation in the ventral prostate gland. J. Biol. Chem. *279*, 1310–1322.

Nascimento, E.M., Cox, C.L., MacArthur, S., Hussain, S., Trotter, M., Blanco, S., Suraj, M., Nichols, J., Kübler, B., Benitah, S.A., et al. (2011). The opposing transcriptional functions of Sin3a and c-Myc are required to maintain tissue homeostasis. Nat. Cell Biol. *13*, 1395–1405.

Nesslinger, N.J., Shi, X.B., and deVere White, R.W. (2003). Androgen-independent growth of LNCaP prostate cancer cells is mediated by gain-of-function mutant p53. Cancer Res. *63*, 2228–2233.

Petersson, M., Brylka, H., Kraus, A., John, S., Rappl, G., Schettina, P., and Niemann, C. (2011). TCF/Lef1 activity controls establishment of diverse stem and progenitor cell compartments in mouse epidermis. EMBO J. *30*, 3004–3018.

Pusapati, R.V., Rounbehler, R.J., Hong, S., Powers, J.T., Yan, M., Kiguchi, K., McArthur, M.J., Wong, P.K., and Johnson, D.G. (2006). ATM promotes apoptosis and suppresses tumorigenesis in response to Myc. Proc. Natl. Acad. Sci. USA *103*, 1446–1451.

Rahl, P.B., Lin, C.Y., Seila, A.C., Flynn, R.A., McCuine, S., Burge, C.B., Sharp, P.A., and Young, R.A. (2010). c-Myc regulates transcriptional pause release. Cell *141*, 432–445.

Reichelt, J., Breiden, B., Sandhoff, K., and Magin, T.M. (2004). Loss of keratin 10 is accompanied by increased sebocyte proliferation and differentiation. Eur. J. Cell Biol. *83*, 747–759.

Rokhlin, O.W., Taghiyev, A.F., Guseva, N.V., Glover, R.A., Chumakov, P.M., Kravchenko, J.E., and Cohen, M.B. (2005). Androgen regulates apoptosis induced by TNFR family ligands via multiple signaling pathways in LNCaP. Oncogene *24*, 6773–6784.

Rosenfield, R.L. (1989). Relationship of sebaceous cell stage to growth in culture. J. Invest. Dermatol. *92*, 751–754.

Rosenfield, R.L., Deplewski, D., Kentsis, A., and Ciletti, N. (1998). Mechanisms of androgen induction of sebocyte differentiation. Dermatology (Basel) *196*, 43–46.

Rosenfield, R.L., Kentsis, A., Deplewski, D., and Ciletti, N. (1999). Rat preputial sebocyte differentiation involves peroxisome proliferator-activated receptors. J. Invest. Dermatol. *112*, 226–232.

Rudolf, E., Rudolf, K., and Cervinka, M. (2011). Camptothecin induces p53-dependent and -independent apoptogenic signaling in melanoma cells. Apoptosis *16*, 1165–1176.

Schirra, F., Suzuki, T., Richards, S.M., Jensen, R.V., Liu, M., Lombardi, M.J., Rowley, P., Treister, N.S., and Sullivan, D.A. (2005). Androgen control of gene expression in the mouse meibomian gland. Invest. Ophthalmol. Vis. Sci. *46*, 3666–3675.

Schirra, F., Richards, S.M., Liu, M., Suzuki, T., Yamagami, H., and Sullivan, D.A. (2006). Androgen regulation of lipogenic pathways in the mouse meibomian gland. Exp. Eye Res. *83*, 291–296.

Schneider, M.R., and Paus, R. (2010). Sebocytes, multifaceted epithelial cells: lipid production and holocrine secretion. Int. J. Biochem. Cell Biol. *42*, 181–185.

Sellheyer, K., and Krahl, D. (2010). Blimp-1: a marker of terminal differentiation but not of sebocytic progenitor cells. J. Cutan. Pathol. *37*, 362–370.

Shah, N.M., Pisapia, D.J., Maniatis, S., Mendelsohn, M.M., Nemes, A., and Axel, R. (2004). Visualizing sexual dimorphism in the brain. Neuron *43*, 313–319.

Sheiness, D.K., Hughes, S.H., Varmus, H.E., Stubblefield, E., and Bishop, J.M. (1980). The vertebrate homolog of the putative transforming gene of avian myelocytomatosis virus: characteristics of the DNA locus and its RNA transcript. Virology *105*, 415–424.

Shenk, J.L., Fisher, C.J., Chen, S.Y., Zhou, X.F., Tillman, K., and Shemshedini, L. (2001). p53 represses androgen-induced transactivation of prostate-specific antigen by disrupting hAR amino- to carboxyl-terminal interaction. J. Biol. Chem. *276*, 38472–38479.

Snippert, H.J., Haegebarth, A., Kasper, M., Jaks, V., van Es, J.H., Barker, N., van de Wetering, M., van den Born, M., Begthel, H., Vries, R.G., et al. (2010). Lgr6 marks stem cells in the hair follicle that generate all cell lineages of the skin. Science *327*, 1385–1389.

Sundberg, J.P., Boggess, D., Sundberg, B.A., Eilertsen, K., Parimoo, S., Filippi, M., and Stenn, K. (2000). Asebia-2J (Scd1(ab2J)): a new allele and a model for scarring alopecia. Am. J. Pathol. *156*, 2067–2075.

Watt, F.M., Frye, M., and Benitah, S.A. (2008). MYC in mammalian epidermis: how can an oncogene stimulate differentiation? Nat. Rev. Cancer *8*, 234–242.

Zouboulis, C.C. (2010). [The sebaceous gland]. Hautarzt *61*, 467–468, 4704, 476–477.

Zouboulis, C.C., Xia, L., Akamatsu, H., Seltmann, H., Fritsch, M., Hornemann, S., Rühl, R., Chen, W., Nau, H., and Orfanos, C.E. (1998). The human sebocyte culture model provides new insights into development and management of seborrhoea and acne. Dermatology (Basel) *196*, 21–31.

Zouboulis, C.C., Seltmann, H., Neitzel, H., and Orfanos, C.E. (1999). Establishment and characterization of an immortalized human sebaceous gland cell line (SZ95). J. Invest. Dermatol. *113*, 1011–1020.

Cell PRESS Open ACCESS

EXTENDED RESULTS

MYC and AR Antibody Validation: Figure S1

Endogenous c-MYC was expressed by differentiated and basal cells in the IFE and SG (Figure S1A). The highest expression was in IFE keratinocytes that were exiting the basal layer (Figure S1H), basal sebocytes (Figure S1I), and the bulb and matrix cells of anagen HFs (Figures S1J and S1K). *K14MycER* transgenic mice showed strong staining in the basal layer of the IFE, basal sebocytes and HF outer root sheath (Figures S1B–S1D), all regions of K14 expression. The same areas were positively labeled with an antibody to the ER domain of the MYCER fusion protein (Figures S1E–S1G). Staining for MYC or ER in *K14MycER* epidermis was similar in acetone and 4OHT treated skin, consistent with the fact that MYCER is expressed in the presence or absence of 4OHT (Arnold and Watt, 2001). The proportion of cells with nuclear MYC increased on addition of 4OHT (compare Figures S1C and S1D and Figures S1F and S1G), as previously shown in keratinocytes cultured from *K14MycER* mice (Berta et al., 2010).

c-MYC antibody specificity was confirmed by immunolabelling epidermis of WT and *K14MycER* mice and epidermis lacking c-MYC. *Myc null* epidermis was generated by topical application of 4OHT to *K14-CreER* x *Myc flox/flox* mice, kindly provided by Elisabete Nascimento and Michaela Frye (Nascimento et al., 2011) (Figures S1L and S1M).

AR-TFM mice lack functional AR expression, owing to a spontaneous single base pair change leading to a frame shift, premature stop codon and nonsense mediated RNA decay of *Ar* mRNA. A small N-terminal fragment of the AR may still be expressed in these mice at low levels but lacks nuclear function (Gaspar et al., 1991). The AR antibody used in this study is raised to the N-terminus of the AR and detected nuclear AR in the lower sebaceous gland in mice with a functional *Ar* allele, such as the *AR-Shah* reporter mouse strain (Figure S1N), but not in *AR-TFM* mice (Figure S1O). At low frequency, the N-terminal AR fragment was occasionally detected as faint peri-nuclear speckling in sebocytes of in *AR-TFM* mice (Figure S1P).

Further Characterization of K14MycER Mice: Figures S2A–S2G

The design of experiments involving topical application of chemical compounds is illustrated in Figure S2A. Figure S2B demonstrates how the size of the differentiated sebocyte compartment in each sebaceous gland was determined by measuring the cross-sectional area (marked by dashed yellow lines) occupied by pale pink sebocytes within each SG. Figure S2C demonstrates the effects on Ki67 labeling of treating *K14MycER* mice and WT mice with low or high dose 4OHT for 4 days and is quantified in Figure 2N.

The *Ar* promoter is detected by ChIP of MYC in *K14MycER* epidermis (Nascimento et al., 2011) and MYC can regulate *AR* expression and activity in the prostate (Nadiminty et al., 2012). As we observed an increase in the number of AR expressing sebocytes upon low dose MYC activation in K14MycER mice, we examined whether *Ar* mRNA expression in back skin was increased. There was no statistically significant change under the conditions tested (Figure S2D), in contrast to the increase in mRNA levels of the proliferation marker *Pcna* and the sebocyte lineage marker *Keratin 7* (*K7*) (Figures S2F and S2G). As 4OHT can inhibit Estrogen Receptor α, a positive regulator of the *AR* gene (Blanchere et al., 1998), we tested if this was the case in skin. In WT mice we did see a small reduction in *Ar* expression but this was not statistically significant (Figure S2E).

Effect of MYC on AR Activity: Figure S2H

To examine whether MYC activation modulated AR signaling, we performed AR-reporter luciferase assays in the human sebocyte line SebE6E7 that was immortalized by introduction of *HPV16 E6* and *E7* genes (Figure S2H) (Lo Celso et al., 2008). SebE6E7 cells exhibited low background AR reporter activity in the presence of DMSO, exogenous testosterone and the anti-androgen casodex, indicating that endogenous AR activity on androgen response elements was low (Figure S2H). However testosterone strongly increased AR activity in AR-transfected sebocytes and this increase was reduced by the addition of casodex (Figure S2H), confirming the pro and anti-androgen function of testosterone and Casodex, respectively.

SebE6E7 cells transfected with *Myc* alone showed no changes in AR activity in the conditions tested. However when the *Ar* and *Myc* were co-transfected, MYC caused a reduction in AR activity in response to testosterone. This indicates that MYC can repress AR signaling in some contexts (Figure S2H). In contrast, in cells co-transfected with *Ar* and *Myc*, MYC promoted AR activity in the presence of Casodex and testosterone (Figure S2H). This may reflect context-dependent recruitment of transcriptional co-regulators to the AR by MYC.

Effects of Casodex on K14MycER Mice: Figure S3

Casodex is a clinically validated anti-androgen used to treat human prostate cancer. It acts in a variety of ways, such as forming inactive, DNA-bound AR transcriptional complexes, competing for AR binding with androgen and preventing heat-shock protein dissociation (Masiello et al., 2002). Since expression of the AR peaked at the onset of sebocytes differentiation but declined during later stages (Figure 1), we treated *K14MycER* mice for 4 days with a low dose of casodex to see how differentiation was affected.

Daily low dose casodex treatment (4x2mg) in combination with a high dose 4OHT did not promote sebocyte differentiation in *K14MycER* mice, confirming that casodex had no agonist properties when combined with MYC activation in vivo (Figures S3A and S3C). However, when combined with a low dose of 4OHT, daily casodex treatment led to enlargement of the SG differentiation compartment (Figures S3B and S3C). This was not due to increased proliferation (data not shown) but rather to enlargement of individual sebocytes at the mid-phase of differentiation (Figure S3D, brackets), reflecting increased lipid accumulation. Late stage differentiation was unaffected (Figure S3E), as there was no increase in the number of mature sebocytes expressing IVL or BLIMP1.

When 4x2mg casodex treatment was combined with 4x2mg testosterone and a high dose of 4OHT sebocytes differentiation was stimulated to a greater extent than with testosterone alone (Figures S3F and S3G). We interpret this result as testosterone stimulating the onset of differentiation by promoting AR-activity in early stages of maturation, while next casodex enhanced late stage differentiation by inhibiting the AR (Figure S3H).

When low dose 4OHT *K14MycER* mice were treated with a higher dose of casodex (4x8mg) for 4 days, SG differentiation was impaired, mimicking the phenotype of *K14MycER AR-TFM* mice. The block in differentiation was overcome by addition of testosterone (Figures S3I and S3J). Application of 4x8mg casodex to WT mice or *K14MycER* mice in the absence of 4OHT did not affect skin histology, nor activate p53 signaling (data not shown).

Our results indicate that in undifferentiated sebaceous gland cells, the AR functions as a differentiation enhancer by promoting cell cycle exit and triggering initial lipid accumulation. However, the AR inhibits the later stages of maturation. Our findings may explain some of the conflicting effects of androgens on sebocyte growth, proliferation and differentiation reported in vitro and in vivo (Akamatsu et al., 1992; Zouboulis, 2010).

Sebaceous Gland Hyperplasia in K14MycER Mice: Figure S4

Low dose treated *K14MycER* mice showed SG enlargement after 4 days, which progressed to SG hyperplasia by 8 days. While the overall differentiation compartment was expanded, this coincided with a partial reduction in AR activity, reduced FASN expression, and accumulation of sebocytes at the top of the gland, suggesting decreased turnover (Figures S4A–S4D). To test its functional significance, we treated mice with daily doses of 2mg testosterone. There was no effect for the first 4 days, which indicates that endogenous AR activity was maximal (Figure S4G). However at 8 days testosterone administration prevented the reduction in AR activity and significantly reduced SG hyperplasia (Figures S4E and S4G). Proliferation of SG progenitors and maturing sebocytes was reduced (Figures S4F and S4H). These data collectively suggest that AR has an anti-proliferative function. Whether human SG hyperplasia is also linked to androgens is debated (Plewig and Kligman, 1978; Pochi et al., 1979; Tagliolatto et al., 2011).

Effects of Inducing p53: Figure S5

To test if p53 activation disrupted SG homeostasis, male *K14MycER* mice where given a low dose of 4OHT and challenged with daily administration of the pro-apoptotic drug camptothecin for 4 days. Some mice also received daily testosterone doses. Camptothecin affected the whole epidermis, but in particular impacted the SG lineage and reduced the SG differentiation compartment (Figure S5C). Camptothecin accelerated clearance of DNA damaged cells marked by γ-H2AX (Figure S5D), increased p53 activity (Figure S5E) and promoted caspase-3 cleavage (Figure S5G). Topical application of camptothecin also triggered reduction of nuclear AR-positive and FASN-positive sebocytes (Figure S5F). Testosterone administration had a modest protective effect by offsetting SG shrinkage, reducing cleaved capase-3 induction, promoting SG AR activity and increasing FASN expression. Testosterone also appeared to decrease induction of p53 throughout the epidermis. These data confirm that an apoptotic level of p53 activation impairs maintenance of the SG lineage by impacting both proliferation and differentiation, and that the AR has a significant role in antagonising the effect of p53 activation.

EXTENDED EXPERIMENTAL PROCEDURES

Transgenic Mice

The following additional strains were used in this analysis: *p53null* mice (B6.129S2-Trp53 tm1Tyj/J; Jackson Laboratory 002101), *AR-Shah* mice (B6.129S-Artm1Rax/ShahJ; Jackson Laboratory 012374) and *Eda (Ta-6J)* x *AR-TFM* mice (B6.Cg-Aw-J EdaTa-6J +/+ ArTfm/J; Jackson Laboratory 001809). *p53null* and *AR-Shah* strains were first crossed onto a C57/Bl6 x CBA F1 background to match our *K14MycER* mice prior to further breeding. *AR-Shah* and *Eda (Ta-6J)* x *AR-TFM* mice were first crossed to create a compound strain of AR-Shah and AR-TFM and bred onto a near C57/Bl6 x CBA F1 background, before further crossing with *K14MycER* mice.

The *AR-Shah* allele contains an *IRES* Alkaline phosphatase *IRES* LacZ reporter cassette knocked into the *Ar* locus following the stop codon (Shah et al., 2004) and was used to track the *AR-TFM* allele by exclusion. Under our breeding strategy, male mice were born with either the AR-competent *AR-Shah* gene or AR-functionless *AR-TFM* gene. The *AR-Shah AR-TFM* strain was further crossed with *K14MycER* and *K14MycER p53null* mice. These mice were 53.125% C57/Bl6 46.875% CBA and 51.56% C57/Bl6 48.44% CBA respectively.

Experiments were performed on male and female mice that were typically aged 7-9 weeks corresponding to the telogen phase of the hair growth cycle (Müller-Röver et al., 2001). However, *K14MycER AR-TFM* and *K14MycER p53null AR-TFM* mice were treated at 6-6.5weeks, as they enter telogen prematurely (Naito et al., 2008). Any mice that strongly re-entered anagen during the course of an experiment were excluded from analysis.

As reported previously, no significant effects of gender on the phenotype of 4OHT treated *K14MycER* mice were observed (Arnold and Watt, 2001; Berta et al., 2010). This is consistent with observations that the skin is a site of androgen synthesis regardless of gender (Chen et al., 2006, 2010). For *AR-Shah AR-TFM* strains, *AR-Shah/Y* true males, homozygous *AR-Shah* females and *AR-TFM/Y* feminised males were used for analysis.

The biological activity of topical applications of 4OHT, testosterone and camptothecin to skin has been reported previously (Berta et al., 2010; Gao et al., 1996a, 1996b; Kao et al., 1985). Topical application of anti-androgens is also a well-established procedure (Moguilewsky and Bouton, 1988). In addition to 4OHT, some mice received daily topical doses of 100 μl 10% DMSO or 0.5 or 1mg camptothecin (Campto, Sigma-Aldrich C9911) in 100 μl 10% DMSO. Camptothecin was insoluble and applied as an emulsion.

Immunofluorescence and Immunohistochemical Labeling

Primary antibodies are listed in Table S1. Secondary antibodies used at 1:600 dilution were Alexa-Fluor 488, 555, 594, or 647-conjugated goat anti-rabbit, anti-mouse or anti-rat IgG or donkey anti-rabbit, anti-mouse, anti-goat or anti-rat IgG (Invitrogen Molecular Probes). Secondary antibodies used at 1:200 dilution were donkey polyclonal anti-Rat IgG-H&L pre-absorbed-Dylight 488 (Abcam ab102260), donkey polyclonal anti-mouse IgG-H&L pre-absorbed-Dylight 649 (Abcam ab98797) and donkey polyclonal anti-goat IgG-H&L pre-absorbed-Dylight 549 (Abcam ab96936). Other secondaries were Streptavidin-Alexa-Fluor 488 (Invitrogen Molecular Probes S11223), diluted 1:250, and rabbit polyclonal anti-mouse-Biotin (Abcam ab97044), Biotin-SP-AffiniPure Donkey Anti- Rabbit IgG (H^+L) (Jackson ImmunoResearch laboratories Inc 711-065-152) used at 1:300 dilution from a 0.65mg/ml stock solution.

Samples of mouse back skin were fixed overnight in 4% paraformaldehyde or 10% neutral buffered formalin and stored in 70% ethanol prior to embedding in paraffin or else frozen in OCT compound (Miles) for horizontal whole mounts (Driskell et al., 2011b). Paraffin sections were dewaxed in xylene 2x for 5mins and sequentially washed with 100% Ethanol 2x 3mins, 96% Ethanol 1x1min, 80% Ethanol 1x1min, distilled water 2x1min, then placed in PBS. Dewaxed sections were transferred to a rice cooker and boiled for 20 min in standard Sodium Citrate Tribasic pH6 buffer. Sections were then cooled for 20mins in PBS, and blocked/permeabilized in 10% Bovine or Goat Serum, 1% BSA, 0.1% Triton X-100, with 1 drop of Fish Scale gelatin (per 20ml) in PBS for 10-20 min. Primary antibodies were applied in 10% Bovine or Goat Serum, 1% BSA+ drop of Fish Scale gelatin in PBS without Triton X-100, for 2 hr at room temperature. Following 3 washes in PBS, secondary antibodies and 40-6-diamidino-2-phenylindole (DAPI), (in same buffer as primary) were incubated with sections for 1.5-2 hr at room temperature. After 3 washes in PBS and 1 wash in distilled water sections were mounted in Slow Fade Gold reagent (Invitrogen).

The procedure for automated immunohistochemistry was to dewax and rehydrate sections on a Leica ST 5020 and then stain sections on a Leica Bond Max or Ventana Discovery. On the Leica Bond Max p53 was labeled with anti-p53 (DO-7) followed a 30' citrate treatment, and AR was labeled with anti-AR (AR27) followed by a 20' EDTA treatment using the Leica Bond Polymer system (DS9800). AR was detected with anti-AR (N-20) followed by a 20' EDTA treatment, and Ki67 with anti-Ki67 (Tec-3) followed by a 20' citrate pre-treatment, performed using Vision Biosystems Bond Intense R biotin/streptavidin Detection system (DS9263). Signals were enhanced by 10' incubation with Bond DAB Enhancer (AR9432-Lecia). All antigen retrieval was performed at 100°C.

The Ventana Discovery system was used for labeling sections with anti-c-MYC, anti-p53 (CM5) and anti-Ki67 (SP6) as described previously (Driskell et al., 2011a; Nascimento et al., 2011). Mouse on mouse FASN staining was performed with a modified protocol whereby a mixture of non-immune mouse IgG1 (Abcam ab81032, 1:100) and non-immune rabbit IgG (SCBT sc-2027, 1:50) was applied during the first antibody incubation. Mouse anti-FASN antibody (1:50) was pre-complexed with rabbit anti-mouse-Biotin (1:250) for a minimum of 15minutes in 1xPBS at room temperature, and any remaining unbound secondary antibody was sequestered by addition of an excess of non-immune mouse IgG (1:100).

Microscopy was performed with a Leica SP5 confocal, Zeiss Axiophot or Zeiss AxioImager M2 fluorescent microscope, using 10x and 20x objectives.

qRT-PCR

0.25-0.5cm^2 pieces of back skin were collected, snap frozen and stored in liquid nitrogen until use. All surfaces and equipment were cleaned with RNase Zap. Samples were placed into round-bottom tubes containing 1.5ml Trizol and homogenized using a polytron (setting 11-15), then transferred into 2ml RNase-free tubes and placed on ice until all samples had been processed. Between samples, the polytron was washed sequentially with 1x9ml DEPC water, 1x9ml 70% Ethanol in DEPC water, 1x9ml 1M NaOH in DEPC water and 3x9ml DEPC water, on setting 15. Samples were incubated at RT for 5′ and then 300ul of BCP reagent was added. Samples were capped and shaken vigorously for 15 s until mixed, and left to separate for an additional 3′ at RT. Samples were next centrifuged for 15' at 12,000 g at 4°C and the aqueous layer transferred to fresh RNase-free tubes. 750ul Isopropanol was added and mixed by inversion then incubated for 10' at RT before being centrifuged for 10' at 12,000 g at 4°C. The supernatant was discarded and the pellet washed in 1ml 70% Ethanol in DEPC, then centrifuged for 1' at 12,000 g at 4°C. The supernatant was again discarded, samples were again centrifuged for 1' at 12,000 g at 4°C and the last traces of ethanol were removed. Samples were left to dry for 10' in a fume hood before addition of 50ul nuclease-free water. Samples were incubated for 10' at 55°C to resuspend RNA. RNA concentration was determined using a nanodrop and preparations stored at −80°C until use. RNAase-free plugged tips were used throughout.

2 μg of RNA was collected and treated with RQ1 DNase (Promega M610A) according to the manufacturer's protocol. cDNA was prepared using Superscript III First-Strand Synthesis SuperMix for qRT-PCR Kit (Invitrogen 11752-250). For qRT-PCR 20 μl reactions were established in 96well plate format, each containing 1 μl of probe, 1 μl cDNA, 8 μl nuclease-free water and 10 μl of 2x Taqman Fast universal reaction master mix (Applied Biosystems). Reactions were performed on a StepOne Real-time PCR machine (Applied Biosystems) using a quick-start protocol. Quantitation was performed using the ΔCT method and normalized relative to GAPDH. Taqman probes (Applied Biosystems) were as follows: *Gapdh* 4352932-1005035, *Ar* Mm00442688_m1, *Keratin 7*

Mm00466677_m1, *Fasn* Mm00662319_m1, *Ki67* Mm01278617_m1, *Pcna* Mm00448100_g1, *Pparγ* Mm011843222_m1, *Nucleolin* Mm01290591_m1, *p53* Mm01337166_mH, *pan p63* Mm00495788_m1, and *pan p73* Mm00660220_m1.

Generation of Plasmids

pRetroQ-mCherry, a mammalian mCherry expression construct, was cloned by excision of mCherry from pmCherry-N1 (Clontech) and exchange with AcGFP in pRetroQ-AcGFP-N1 (Clontech). pRetroQ-Flag-AR-T2A-mCherry, a mammalian dual non-covalently-linked AR and mCherry expression construct, was cloned by PCR addition of an N-terminal Flag-tag and C-terminal T2A linkage sequence to mouse AR cDNA using template pENTR233.1-AR (Clone ID 100016477, Source Bioscience) with product subcloned into pRetroQ-mCherry. pCDNA4-Myc, a kind gift from Dr. Alastair Lamb (Neal laboratory, Cambridge Research Institute, Cancer Research UK), was generated by excision of Myc from pBABEpuro c-mycER (Littlewood et al., 1995) and ligation into pCDNA4 (Invitrogen). Plasmid DNA was prepared using QIAGEN products.

AR Reporter Luciferase Assays

SebE6E7 immortalized human sebocytes were cultured in serum and growth factor supplemented Sebomed medium (Biochrom) as previously described (Lo Celso et al., 2008). SebE6E7 sebocytes were seeded in 48-well plates at a density of 4-5x10^4 cells per well and allowed to adhere overnight. Adherent sebocytes were transfected with 0.2 μl Xfect (Clontech), mixed with 0.24 μg pRetroQ-mCherry (parent-vector) or pRetroQ-Flag-AR-T2A-mCherry (AR) and 0.24 μg pCDNA4 (parent-vector) or pCDNA4-Myc (Myc), with 0.2 μg pTA-Luc (background control, LR0000, Panomics) or pAR-Luc (AR-reporter, LR0007, Panomics) firefly luciferase plasmids and 0.02 μg pCMV-RL Renilla luciferase reporter (Promega) for 4 hr according to the manufacturer's protocol. Total DNA was maintained at 0.7 μg per well by inclusion of parent-vectors as needed.

Sebocytes were incubated in supplemented Sebomed medium overnight, then transferred to medium containing 20 μM testosterone (T1500, Sigma-Aldrich) and/or 10 μM casodex (Bicalutamide, B9061, Sigma-Aldrich) or DMSO-vehicle for 24 hr. Testosterone and casodex stocks in DMSO were prepared at 1000x. Cells were lysed and assayed using the Promega dual luciferase reporter kit according to the manufacturer's instructions. Data were collected on a Glomax luminometer (Promega). Luciferase values were normalized by dividing with Renilla luciferase activity values to adjust for transfection efficiency. Each experiment was performed in triplicate and values averaged for each condition. pAR-Luc data are expressed as fold change over values obtained from equivalent conditions with pTA-Luc to adjust for global transcriptional changes and highlight Androgen Response Element-dependent changes. Changes are expressed relative to vehicle (defined as 1) for each transfection combination. This experiment was performed 5 times with independent cell populations.

SUPPLEMENTAL REFERENCES

Blanchere, M., Berthaut, I., Portois, M.C., Mestayer, C., and Mowszowicz, I. (1998). Hormonal regulation of the androgen receptor expression in human prostatic cells in culture. J. Steroid Biochem. Mol. Biol. *66*, 319–326.

Driskell, R.R., Juneja, V.R., Connelly, J.T., Kretzschmar, K., Tan, D.W., and Watt, F.M. (2011b). Clonal growth of dermal papilla cells in hydrogels reveals intrinsic differences between Sox2-positive and -negative cells in vitro and in vivo. J. Invest. Dermatol. *132*, 1084–1093.

Gao, X.M., Perchellet, E.M., Davis, A.W., Newell, S.W., Chen, G., Hua, D.H., and Perchellet, J.P. (1996a). Camptothecin post-treatments inhibit the biochemical events linked to the tumor-promoting component of carcinogenesis in mouse epidermis in vivo. Int. J. Cancer *66*, 496–505.

Gao, X.M., Perchellet, E.M., Davis, A.W., Newell, S.W., Hua, D.H., and Perchellet, J.P. (1996b). Characterization of the antitumor-promoting activity of camptothecin in SENCAR mouse skin. Carcinogenesis *17*, 1141–1148.

Kao, J., Patterson, F.K., and Hall, J. (1985). Skin penetration and metabolism of topically applied chemicals in six mammalian species, including man: an in vitro study with benzo[a]pyrene and testosterone. Toxicol. Appl. Pharmacol. *81*, 502–516.

Littlewood, T.D., Hancock, D.C., Danielian, P.S., Parker, M.G., and Evan, G.I. (1995). A modified oestrogen receptor ligand-binding domain as an improved switch for the regulation of heterologous proteins. Nucleic Acids Res. *23*, 1686–1690.

Masiello, D., Cheng, S., Bubley, G.J., Lu, M.L., and Balk, S.P. (2002). Bicalutamide functions as an androgen receptor antagonist by assembly of a transcriptionally inactive receptor. J. Biol. Chem. *277*, 26321–26326.

Moguilewsky, M., and Bouton, M.M. (1988). How the study of the biological activities of antiandrogens can be oriented towards the clinic. J. Steroid Biochem. *31*(4B), 699–710.

Müller-Röver, S., Handjiski, B., van der Veen, C., Eichmüller, S., Foitzik, K., McKay, I.A., Stenn, K.S., and Paus, R. (2001). A comprehensive guide for the accurate classification of murine hair follicles in distinct hair cycle stages. J. Invest. Dermatol. *117*, 3–15.

Naito, A., Sato, T., Matsumoto, T., Takeyama, K., Yoshino, T., Kato, S., and Ohdera, M. (2008). Dihydrotestosterone inhibits murine hair growth via the androgen receptor. Br. J. Dermatol. *159*, 300–305.

Plewig, G., and Kligman, A.M. (1978). Proliferative activity of the sebaceous glands of the aged. J. Invest. Dermatol. *70*, 314–317.

Pochi, P.E., Strauss, J.S., and Downing, D.T. (1979). Age-related changes in sebaceous gland activity. J. Invest. Dermatol. *73*, 108–111.

Tagliolatto, S., Alchorne, M.M., and Enokihara, M. (2011). Sebaceous hyperplasia: a pilot study to correlate this skin disease with circulating androgen levels. An. Bras. Dermatol. *86*, 917–923.

Cell PRESS
Open ACCESS

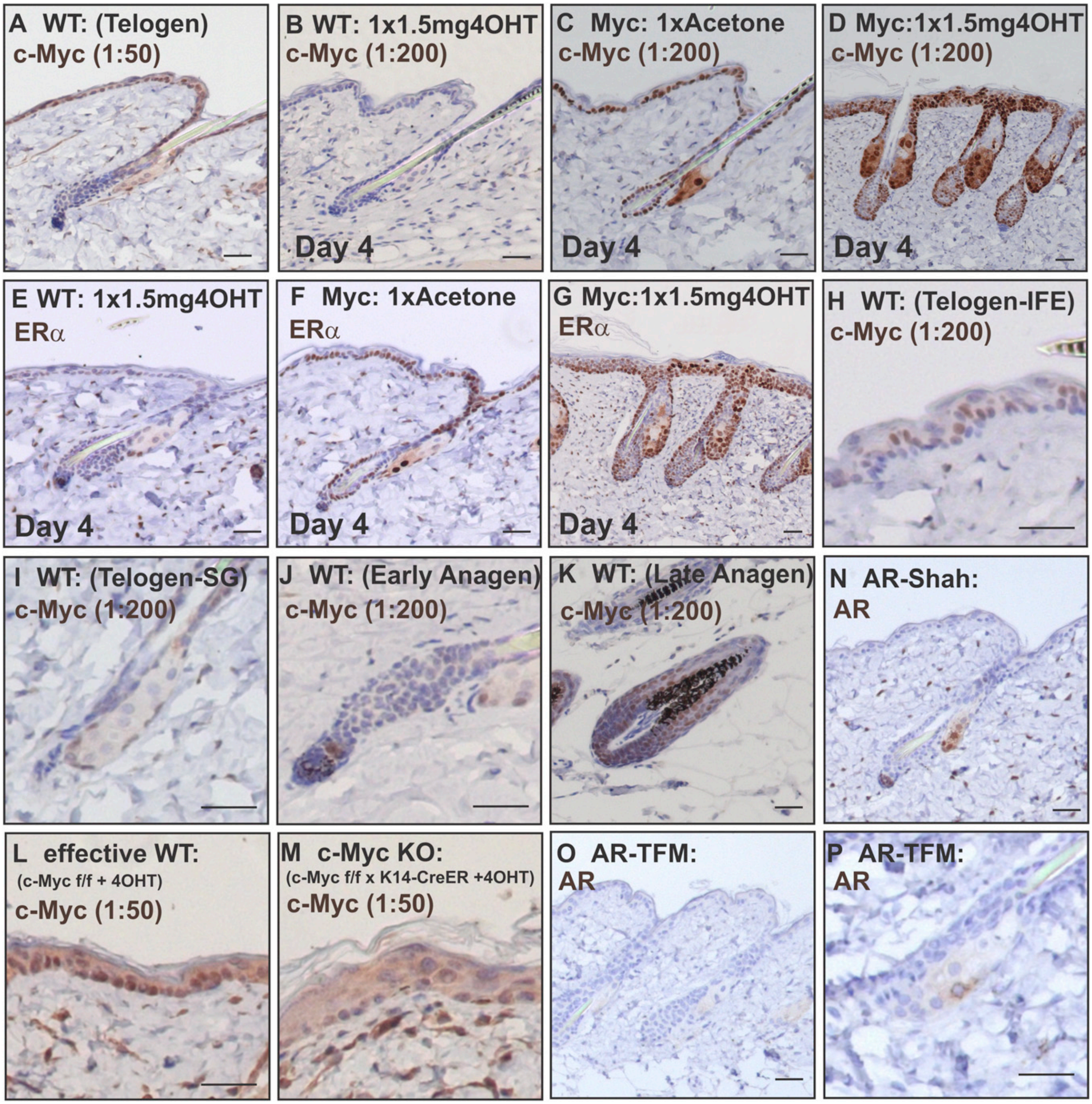

Figure S1. MYC and AR Antibody Validation on Sections of Mouse Back Skin, Related to Figure 1

(A–D and H–M) Labeling with anti-cMYC antibody at the dilutions shown. WT: wild-type; Myc: MycER; c-Myc KO: deletion of *Myc* via *K14CreER*. Homozygous *c-Myc floxed* x *K14CreER* transgenics were treated with 1.5mg 4OHT every second day for a minimum of 21 days.

(E–G) Labeling with ERα antibody to detect MYCER fusion protein.

(N, O, and P) AR antibody labeling on *AR-Shah* and *AR-TFM* mice.

Scale bars 40 μm.

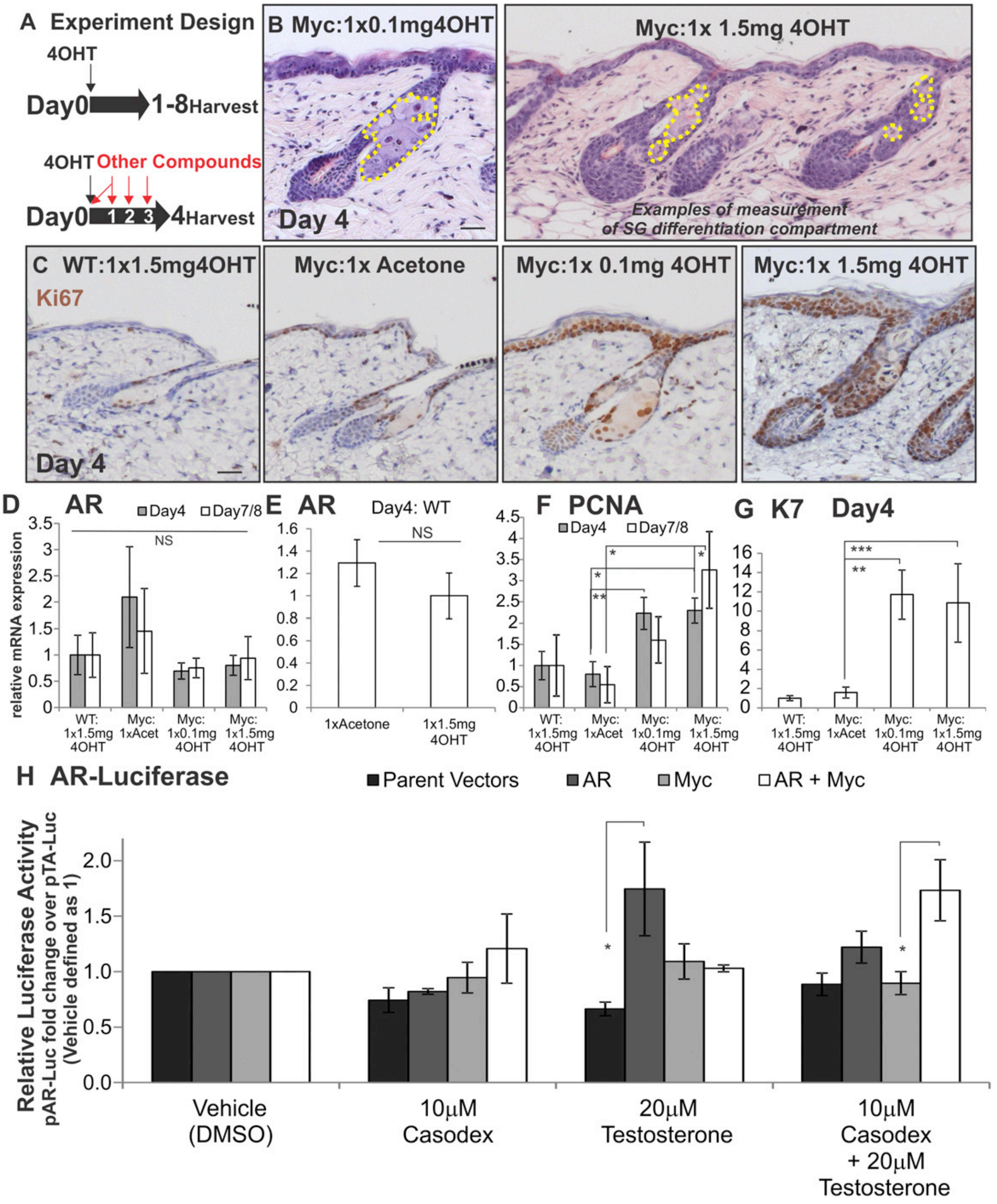

Figure S2. Further Characterization of *K14MycER* Mice and Effects of MYC activation on AR Signaling, Related to Figure 2

(A) Scheme of experimental treatments.

(B) Examples of differentiated SG compartment, (cross-sectional area occupied by differentiated sebocytes within yellow-dashed regions).

(C) Ki67 labeling of *K14MycER* and WT control mice 4 days following treatment, quantified in Figure 2N.

(D–G) qRT-PCR of mRNA levels of *Ar* (D, E), *Pcna* (F), and *K7* (G) relative to *Gapdh*. Error bars represent SEM.

(H) AR-reporter luciferase assays in SebE6E7 human immortalized sebocytes. Data are means of 5 independent experiments ± SEM. NS = not significant, *p < 0.05, **p < 0.01, ***p < 0.005.

Scale bars 40 μm.

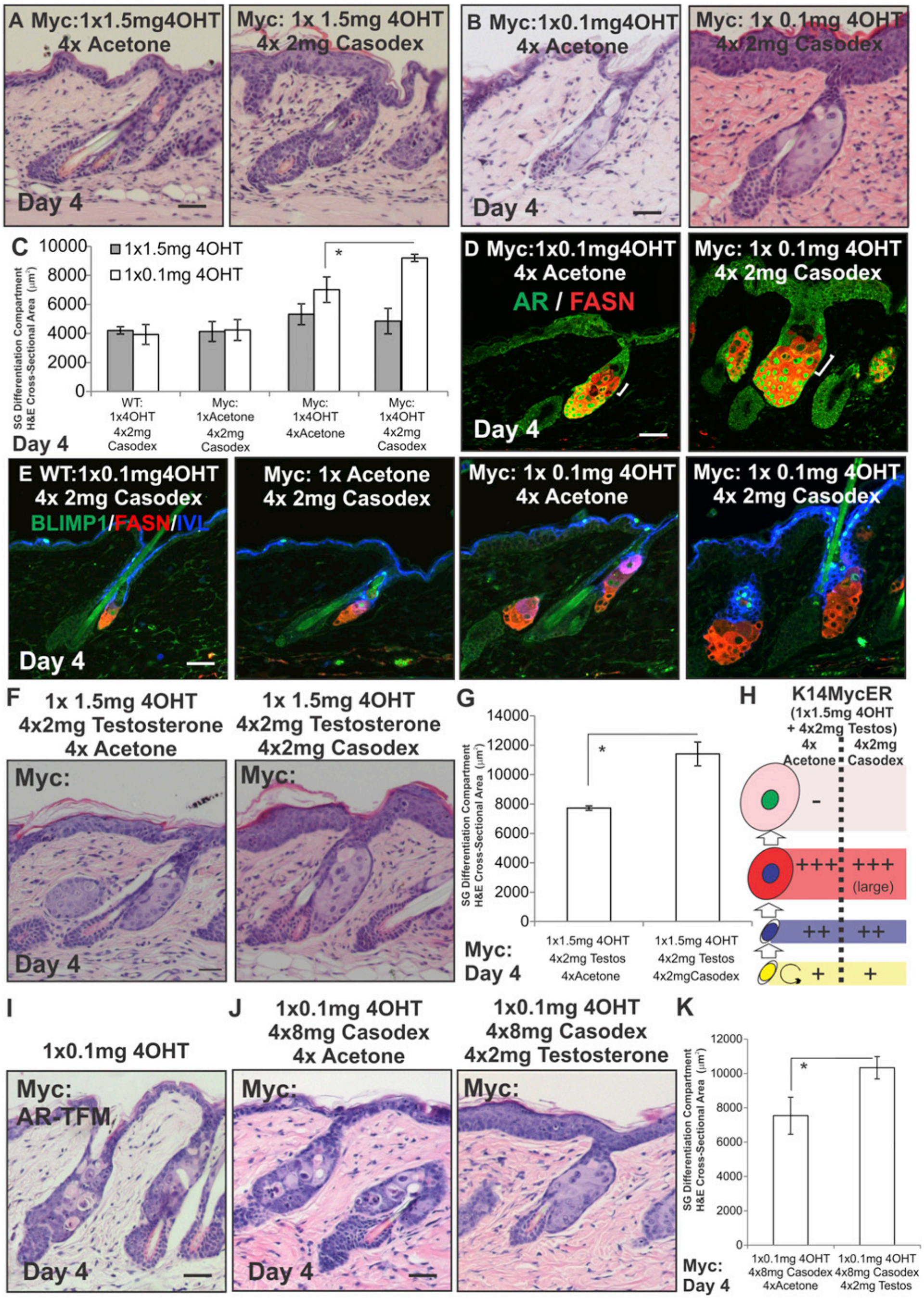

Figure S3. Casodex Treatment of *K14MycER* Mice, Related to Figure 4

(A–K) Back skin of *K14MycER* mice (Myc:) was stained with H&E (A, B, and F) or labeled with the antibodies shown (D, E, I, and J). Skin in (I) was from a cross with *AR-TFM*. White brackets in (D) indicate differences in size of individual sebocytes. (C, G, and K) Quantitation of SG differentiation compartment. (H) Schematic summary of changes relative to WT SG of treating *K14MycER* mice with high dose 4OHT in the presence or absence of casodex. See Figure 1N for stages in sebocytes differentiation. Increases in cell number are represented by +, ++ or +++, according to the strength of the effect. Reduction in cell number is represented by -.

3-5 mice were examined per condition. Error bars represent SEM. NS = not significant, *$p < 0.05$. Scale bars 40 μm.

Cell PRESS
Open ACCESS

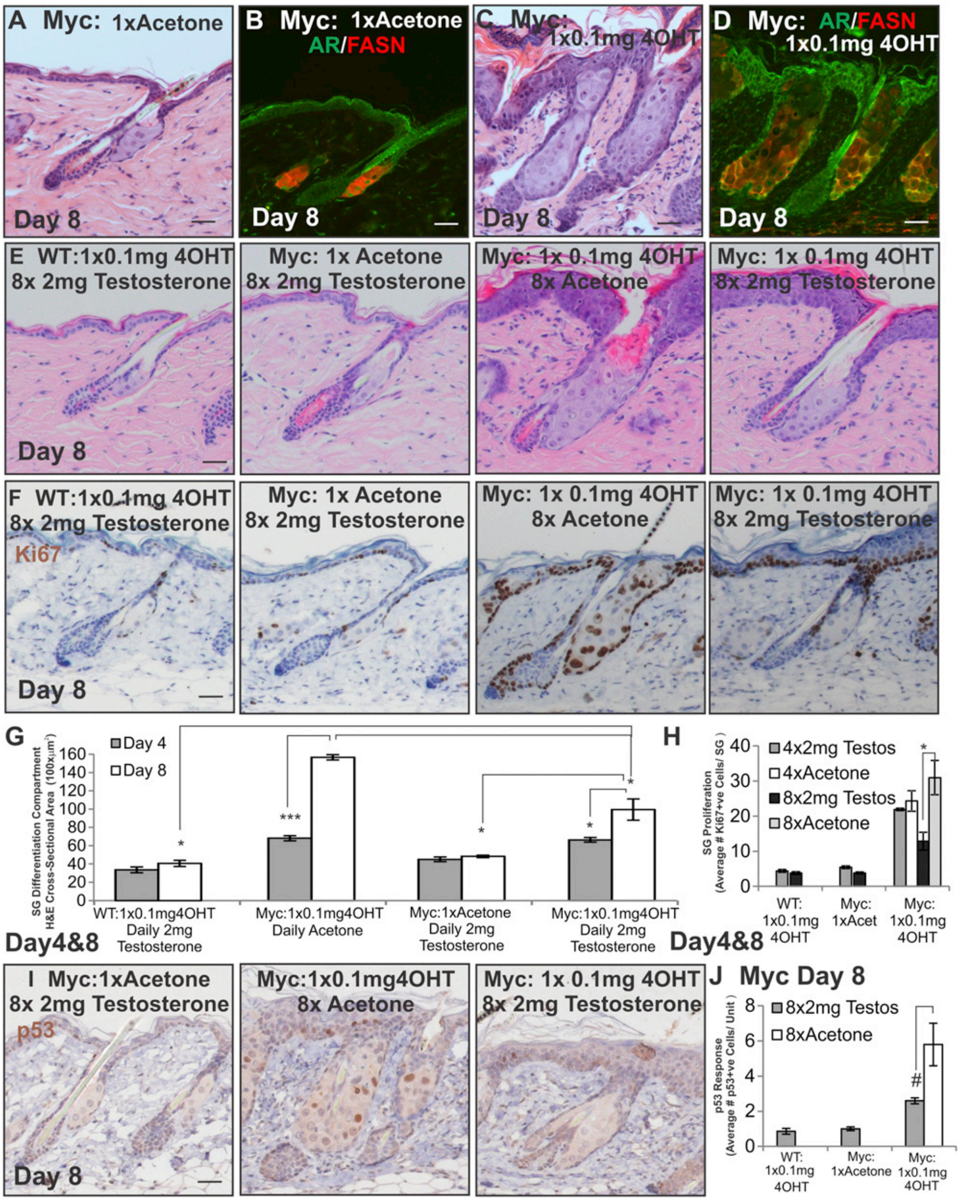

Figure S4. ***K14MycER* Sebaceous Gland Hyperplasia, Related to Figure 4**
(A, C, and E) H&E, (B and D) AR and FASN, (F) Ki67 and (I) p53 staining of wild-type (WT) and *K14MycER* (Myc:) mice.
(G) Quantitation of size of SG differentiation compartment. H) Quantitation of Ki67 positive cells per sebaceous gland.
(J) Quantitation of number of p53 positive sebocytes per unit area of skin.
3 mice were examined per condition, Error bars represent SEM, #p < 0.06, *p < 0.05, ***p < 0.005. Scale bars 40 μm.

Cell PRESS
Open ACCESS

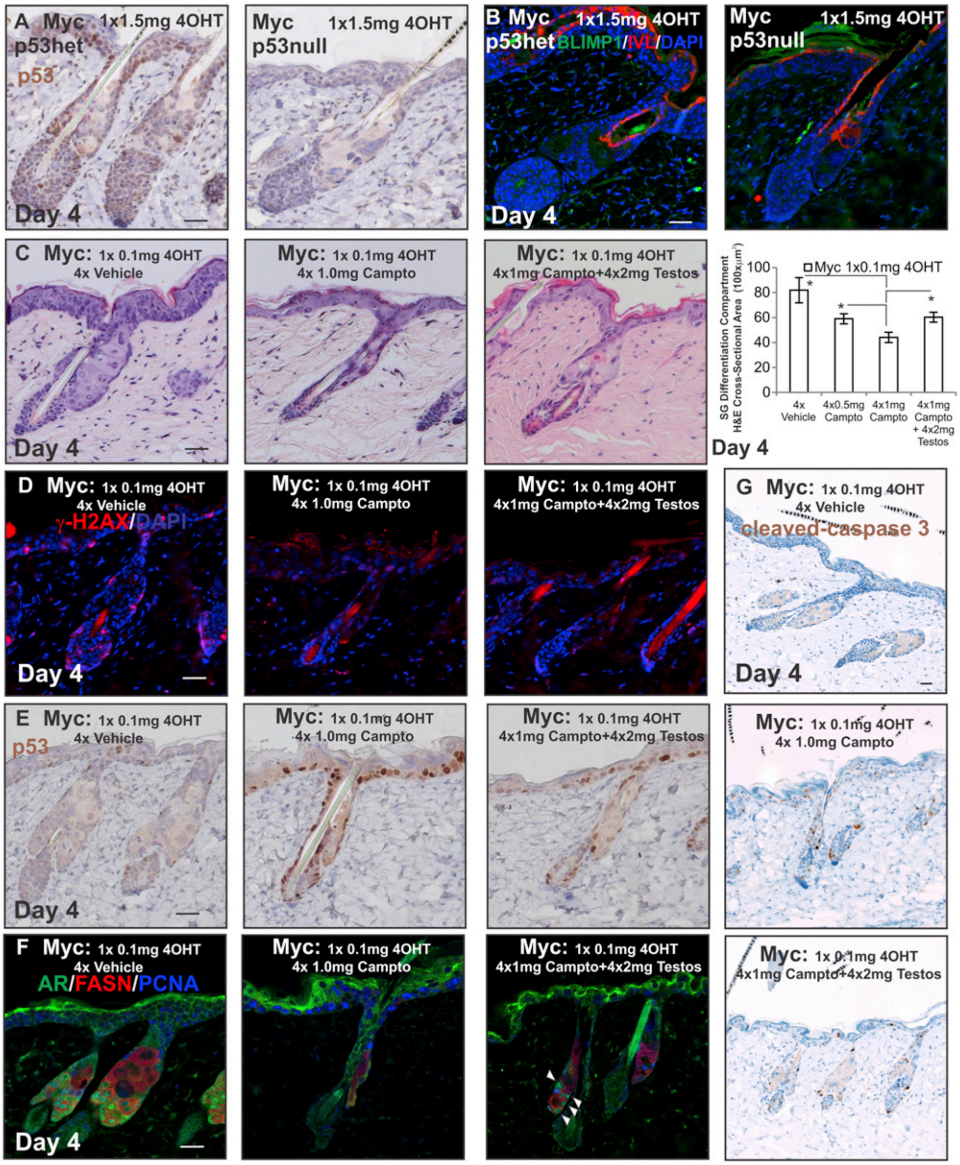

Figure S5. Effect on *K14MycER* Mice of Activating p53 with Camptothecin, Related to Figure 6

(A) Validation of p53 antibody staining.

(B) Immunostaining of BLIMP1 and Involucrin (IVL).

(C) H&E staining. Graph demonstrates quantitation of SG differentiation compartment.

(D) γ-H2AX, (E) p53, (F) PCNA, AR and FASN, (G) Cleaved caspase-3 labeling. White arrowheads indicate AR-positive sebocytes.

3-5 mice were examined per condition. Error bars represent SEM, #$p < 0.06$, *$p < 0.05$, **$p < 0.01$, ***$p < 0.005$. Scale bars 40 μm.
